# The evolution of cyclopropenium ions into functional polyelectrolytes

**DOI:** 10.1038/ncomms6950

**Published:** 2015-01-09

**Authors:** Yivan Jiang, Jessica L. Freyer, Pepa Cotanda, Spencer D. Brucks, Kato L. Killops, Jeffrey S. Bandar, Christopher Torsitano, Nitash P. Balsara, Tristan H. Lambert, Luis M. Campos

**Affiliations:** 1 Department of Chemistry, Columbia University, New York, New York 10027, USA; 2 Joint Center for Artificial Photosynthesis, Lawrence Berkeley National Laboratory, Berkeley, California 94720, USA; 3 Department of Chemical and Biomolecular Engineering, University of California, Berkeley, California 94720, USA; 4 Edgewood Chemical Biological Center, Aberdeen Proving Ground, Aberdeen, Maryland 21010, USA; 5 Materials Sciences Division, Environmental Energy Technologies Division and Joint Center for Energy Storage, Lawrence Berkeley National Laboratory, Berkeley, California 94720, USA

## Abstract

Versatile polyelectrolytes with tunable physical properties have the potential to be transformative in applications such as energy storage, fuel cells and various electronic devices. Among the types of materials available for these applications, nanostructured cationic block copolyelectrolytes offer mechanical integrity and well-defined conducting paths for ionic transport. To date, most cationic polyelectrolytes bear charge formally localized on heteroatoms and lack broad modularity to tune their physical properties. To overcome these challenges, we describe herein the development of a new class of functional polyelectrolytes based on the aromatic cyclopropenium ion. We demonstrate the facile synthesis of a series of polymers and nanoparticles based on monomeric cyclopropenium building blocks incorporating various functional groups that affect physical properties. The materials exhibit high ionic conductivity and thermal stability due to the nature of the cationic moieties, thus rendering this class of new materials as an attractive alternative to develop ion-conducting membranes.

## Abstract (web summary)

Cationic polyelectrolytes have a broad range of applications, including membranes for fuel cells. Here, the authors report a family of cationic polyelectrolytes based on the highly modular cyclopropenium ion building block, which show high ionic conductivity and tunable physical properties.

## Graphical abstract

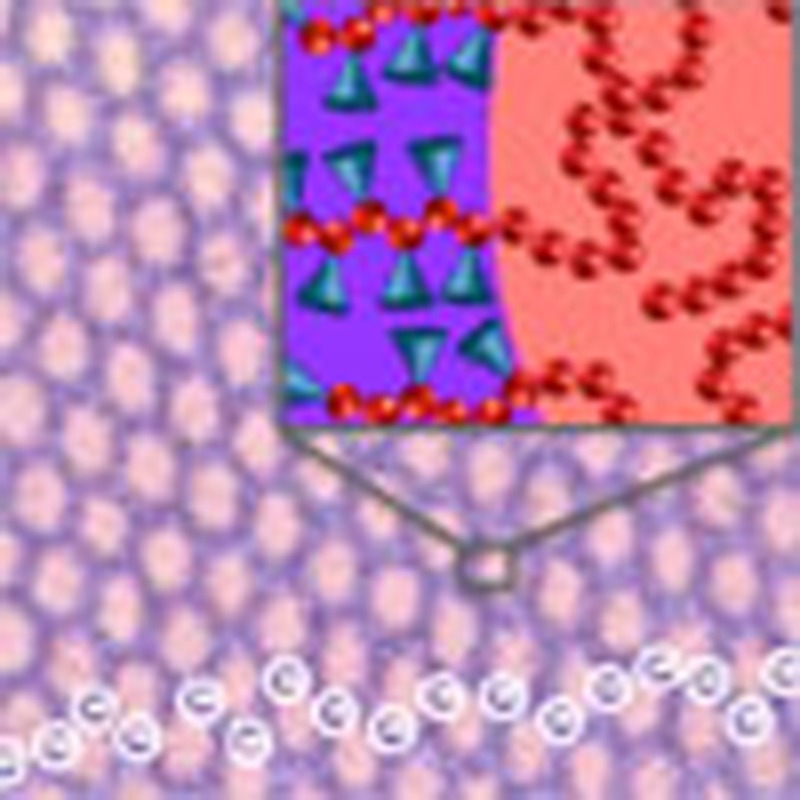

Modularly designed polymeric materials can be engineered to suit a broad range of applications representing an attractive platform for technological advancement^1^. Materials that possess both inherent compositional versatility and ready accessibility via robust and scalable synthetic pathways are of particular import to the field^2^,^3^. In this regard, cationic polyelectrolytes have emerged as a versatile class of materials that have been exploited in a broad array of applications^4^,^5^,^6^, ranging from gene delivery^7^,^8^ to ion-conducting membranes^9^,^10^,^11^, and water purification^12^,^13^. Development in the area of cationic polyelectrolytes has thus far focused on a limited menu of monomeric functionalities, including ammonium, phosphonium, imidazolium, pyridinium and guanidinium ions^14^,^15^,^16^. These heteroatomic systems, while valuable, are application specific and are limited in terms of the ability to finely tune their physical properties. Thus, the identification of new modular cationic polyelectrolytes, with superior characteristics for processing, controllable self-assembly and function, represents an important goal for this field^17^,^18^.

In developing a new family of polyelectrolytes, certain criteria must be met^11^,^13^,^17^ including: (1) thermodynamic stability; (2) ease and scalability of polymerisations by controlled methods; (3) incorporation of accessible chemical handles to allow for diversity and intimate control of physical properties and (4) tunable Coulombic interactions. As an outgrowth of ongoing research efforts, we postulated that polyelectrolytes based on the cyclopropenium ion could satisfy these design criteria, while offering a highly distinct structural architecture and electronic properties. We further recognized that such cyclopropenium-based systems possess unique characteristics that distinguish them from existing cationic polyelectrolytes, namely: enhanced dispersion of the positive charge (compared with ammonium, phosphonium and guanidinium systems) and weaker H-bond donor capacity (compared with imidazolium and pyridinium ions)^19^.

As the smallest of the Hückel aromatics^20^, the cyclopropenium (CP) ion possesses significant stability despite its carbocationic nature (Fig. 1a)^21^,^22^. This remarkable degree of stability may be further enhanced through the incorporation of amino substituents onto the CP ring^23^. Indeed, with p*K*_R+_ values estimated at >13, aminocyclopropenium ions are stable even in strongly alkaline aqueous solutions^24^,^25^. Moreover, thermal decomposition (*T*_dec_) of the tris(dialkylamino)CP chloride salts has been measured at >300 °C^19^, significantly exceeding that of dialkylimidazolium chloride salts (*T*_dec_ ~250 °C)^26^. These unique structural features have already inspired the development of aminocyclopropenium ions for a range of applications, including as metal ligands^27^, organocatalysts^28^,^29^,^30^ and ionic liquids^19^; however, the incorporation of these cations into a polymeric backbone has only led to polymers with unstable CP ions as intermediates^31^. Given the tunable functionality and robust, efficient and orthogonal chemistry characterizing CP ions, it is desirable to exploit them in polymeric materials.

Here, we describe the synthesis and evaluation of a new family of cationic polyelectrolytes. As outlined in Fig. 1b, our vision for the design of the parent ionic monomer includes a polymerisable unit, a spectator group (which could also serve as a functional handle) and four additional modular groups that provide the means to tune the physical properties of the resulting macromolecules. In these initial studies, we elected to focus on styrenic CP monomers (termed, **CPR**) bearing a series of dialkylamino (NR_2_) substituents. Styrene-based monomers can be subjected to various reversible-deactivation radical polymerization strategies^32^,^33^,^34^. We used reversible-addition fragmentation chain transfer (RAFT) polymerization^34^ to assemble homopolymers, statistical copolymers and diblock copolymers of different compositions ranging from 20 to 50 mol % of CP functionality. As will be shown, we demonstrate that macromolecular assemblies of these materials can be used as ion-conducting membranes, and that the physical properties of these assemblies may be tuned through variation of the dialkylamino handles. In addition, we demonstrate that **CPR** monomers undergo a surfactant-free emulsion polymerization with styrene, yielding well defined, sub-100 nm nanoparticles with charged surfaces.

## Results

### Monomer synthesis

**Figure 1:**
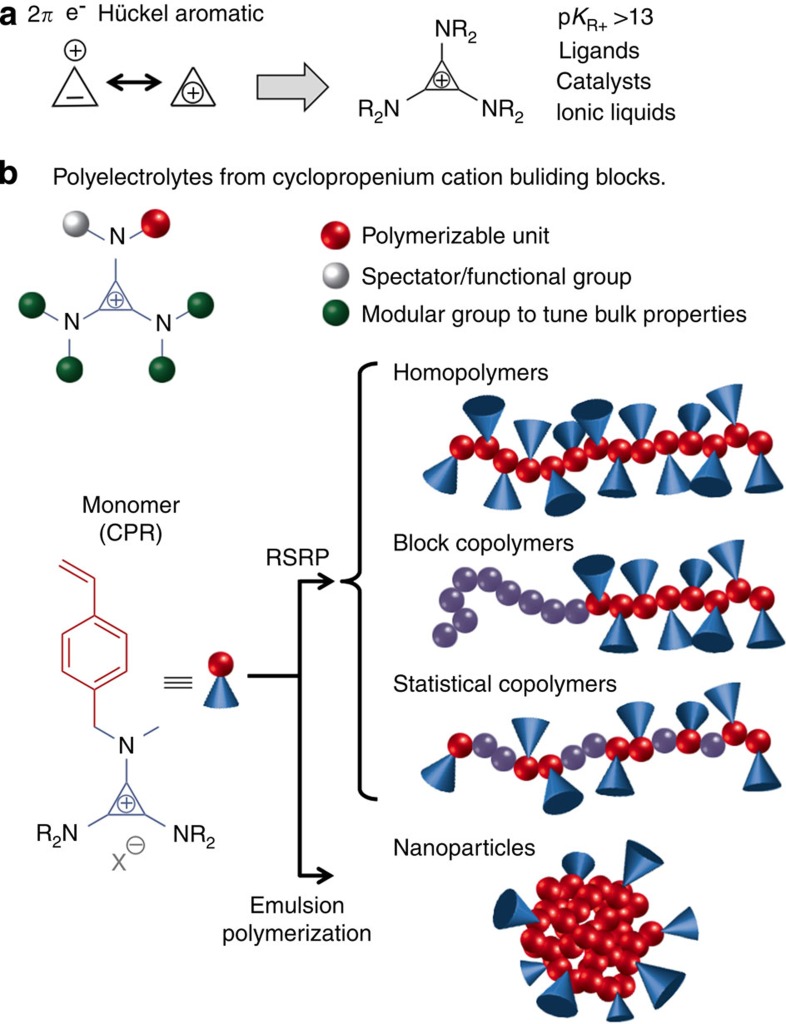
CP ion building blocks. (**a**) Structure of the CP ion, including the dialkylamino groups that can be used to stabilize and vary the application of this diverse building block. (**b**) Types of polyelectrolytes that can be synthesized from CP monomers by reversible-deactivation radical polymerization (RDRP) strategies and emulsion polymerization.

Our exploration of the CP functional group in the context of cationic polyelectrolytes was originally inspired by its ionic liquid properties^19^ and the straightforward elaboration of the CP ion with various functional groups. Thus far, however, there are no reports on the incorporation of this thermodynamically stable carbocation into macromolecules; CP derivatives have only appeared in polymers as transient species^31^,^35^. Derivatives of the CP ion are made from inexpensive reagents and can be easily prepared on a multigram scale under ambient conditions^19^. As robust chemistry is requisite for large-scale production of materials, we devised a viable synthetic strategy *en route* to the polymerisable CP ion monomers. The general approach to synthesise CP ion-containing monomers is based on the facile preparation of asymmetric amino-substituted CP ions (for example, **CPR**, Fig. 1)^36^. This procedure allows us to intimately change functionality while maintaining cationic properties and thermal stability. Notably, synthetic routes to aminocyclopropenium derivatives are modular and highly scalable^22^, with efficiency levels approaching those attained via click chemistry^37^.

**Figure 2:**
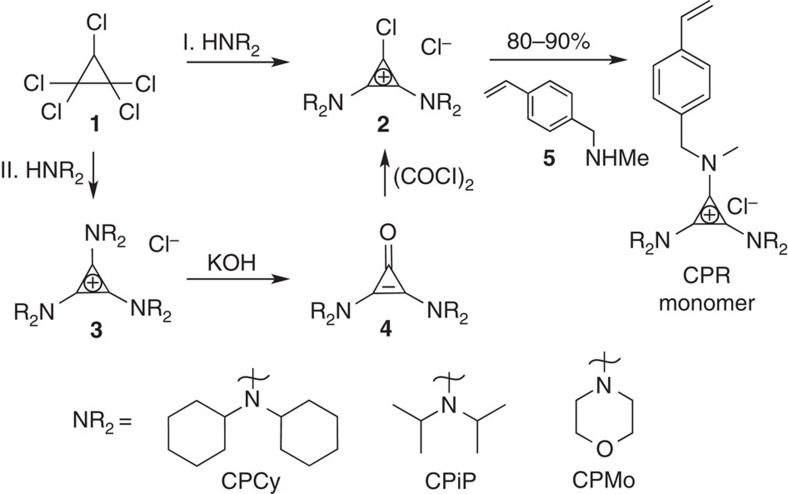
Synthesis of CP monomers for RAFT polymerisations. The monomers **CPCy** and **CPiP** were synthesized by addition of dicyclohexylamine (92%) and diisopropylamine (85%) to **1**, followed by substitution of styrenic-type amine **5** under basic conditions (86% and 88%, respectively). **CPMo** was similarly synthesized. After addition of morpholine to **1**, subsequent hydrolysis and treatment with oxalyl chloride (42%, 3 steps), **5** was substituted to yield **CPMo** (59%).

The preparation of the **CPR** monomers begins with pentachlorocyclopropane (**1**), which is commercially available or inexpensively synthesized in hundred-gram quantities^38^. Reaction of **1** with a secondary amine leads to near-quantitative yields of a corresponding CP cation (**2** or **3**). Thus, amines with high steric hindrance (dicyclohexylamine, Cy and diisopropylamine, iP) add twice to **1**, preventing addition of a third bulky amine and leading directly to **2**. Conversely, less sterically hindered amines, such as morpholine (Mo), add thrice to **1**, resulting in a *tris*-amino CP (**3**). The latter is readily hydrolyzed to its corresponding cyclopropenone in hot, aqueous base, which is subsequently chlorinated to obtain **2**. To underscore the accessibility of these materials, we note that the monomers are obtained by simple purification techniques (see Supplementary Methods for details). Using this process, we prepared multigram quantities of **2** incorporating three different secondary amines, as depicted in Fig. 2 (bottom). Importantly, the chemistry in Fig. 2 is highly amenable to a wide range of nucleophilic secondary amines incorporating a variety of functional groups, including elements of asymmetry. We specifically chose to examine dicyclohexylamine, diisopropylamine and morpholine, given that they differ significantly in their degrees of hydrophilicity and steric hindrance.

The synthesis of the **CPR** monomers from the precursor (**2**) was readily achieved in 10–20 g quantities. The chlorinated 1-position of **2** is highly susceptible to addition of a secondary amine bearing a polymerisable unit, such as compound **5**. A styrene-based polymerisable group was chosen as it is a well-behaved building block in polymer chemistry, and its hydrophobicity relative to the CP ion moiety could stabilize an emulsion of the type used in nanoparticle synthesis. We postulate that other polymerisable moieties would yield functional monomers as well.

### Polymer synthesis and characterization

**Figure 3:**
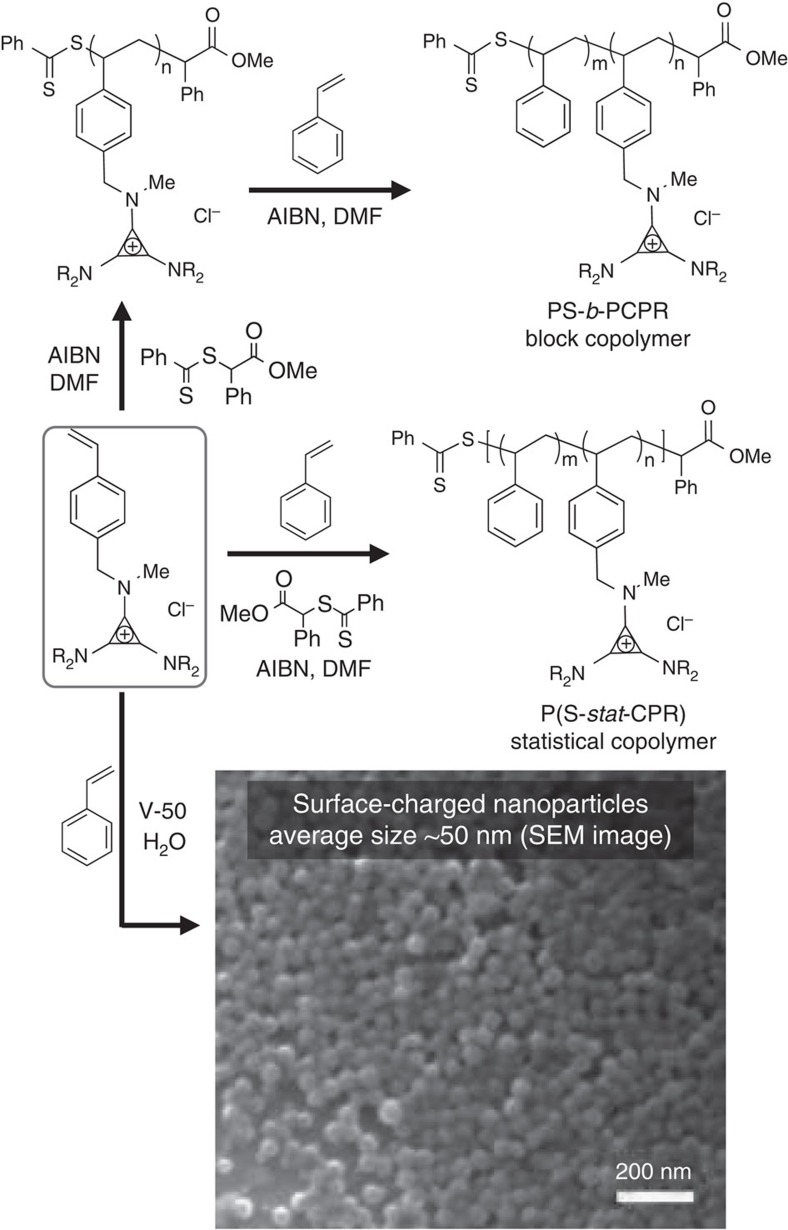
Synthesis of CP-containing polymers. **CPR** is polymerized by RAFT yielding both homopolymers, **PCPR**, and statistical copolymers, **P(S-*****stat*****-CPR)**. **PCPR** is reacted further to form block copolymers **PS-*****b*****-PCPR** of varying styrene content. Nanoparticles are synthesized by surfactant-free emulsion polymerization with styrene using the water-soluble thermal initiator V-50.

The three chosen monomers (**CPCy**, **CPiP** and **CPMo**, Fig. 2) were polymerized by RAFT in multigram quantities yielding linear polymers (**PCPCy**, **PCPiP** and **PCPMo**, respectively, Fig. 3). **PCPCy** was purified through precipitation into 1,4-dioxane from CH_2_Cl_2_ with 88% recovered yield. **PCPiP** was precipitated from acetone or CH_2_Cl_2_ into cold ethyl acetate with 70% recovered yield. Due to their solubility in water, both **PCPiP** and **PCPMo** can be purified by dialysis. Purification of **PCPMo** resulted in a 51% recovered yield. Each of these polymers was isolated as a powder, and **PCPiP** and **PCPMo** were observed to be extremely hygroscopic. Through end-group analysis of the ^1^H nuclear magnetic resonance (NMR) spectra, we calculated the degree of polymerization (DP) and molecular mass of each of the homopolymers (Supplementary Table 1). Due to the cationic nature of the CP groups, polymers (and copolymers) cannot be characterized using size exclusion chromatography eluted with organic solvents, as the polymers adhere to the column. We attempted to characterise the dispersity (*Đ*) of the hydrophilic homopolymers (**PCPiP** and **PCPMo**) on an acetate-buffered aqueous size exclusion chromatography; however, only **PCPMo** successfully eluted owing to its greater hydrophilicity (Supplementary Fig. 1, Supplementary Table 2). The *Đ* of **PCPMo** was determined to be 1.3, but we note that this value may not accurately reflect the DP control, given that the polyelectrolyte may still be interacting with the column as it is eluted. We note that the synthetic accessibility of these various CP-based polymers is straightforward and highly efficient, rivalling that of ammonium, phosphonium and imidazolium polymers^39^,^40^,^41^,^42^.

As expected, a significant relationship was observed between the nature of the amino substituent and the physical properties of the resultant homopolymer. We observed that the decomposition temperature (*T*_dec_), glass transition temperature (*T*_g_) and solubility of the homopolymers varied as a function of substituent (Supplementary Table 1). Through characterization by thermogravimetric analysis we found that the *T*_dec_ of the homopolymers increased as the amino substituents became less sterically hindered. Of particular note, **PCPMo** decomposed at 310 °C, which is comparable to the *T*_dec_ of imidazolium-based polymers^43^. Differential scanning calorimetry was performed to identify the *T*_g_ for the homopolymers, as ion-conducting membranes are frequently melt-processed. Both **PCPCy** and **PCPiP** have no observable *T*_g_ before decomposition, but **PCPMo** exhibited a *T*_g_ of 160 °C. Previous reports have revealed a connection between the nature of the counterion and the accessible temperature window; replacement of the chloride with an alternative, typically bulkier counterion is expected to increase the *T*_dec_ while decreasing the *T*_g_^19^,^43^,^44^. Importantly, however, our preliminary data clearly demonstrate a similar relationship between alkyl chain identity and observed *T*_dec_ and *T*_g_; thus, by modifying the CP substituents, we are able to significantly broaden the temperature window in which these materials are processable, without the need to adjust the counterion. In addition, solubility of **PCPR** homopolymers is highly dependent on amino substituents, again reflecting the influence of building block composition on macromolecular properties. Characterization of the homopolymers, including thermal data and solubility information, is summarized in Supplementary Table 1.

Statistical copolymers were readily synthesized by RAFT, using styrene and **CPR** monomers, **P(S-*****stat*****-CPR)**. When styrene was copolymerized with each monomer in a 1:1 mole ratio, we observed some disparities in the percent incorporation of functional CP monomers in the resulting copolymer. For **CPCy**, **CPiP** and **CPMo,** the degree of incorporation was ~50%, 48%, and 45%, respectively. Further detailed studies with other monomers are underway to uncover insights into reactivity ratios as a function of R-substituents.

Considering the ability to copolymerize styrene and **CPR** monomers, we sought to synthesise cationic nanoparticles based on the **CPiP** monomer via surfactant-free emulsion polymerization. Many traditional strategies rely on the use of surfactants or additional solvents^45^ to obtain sub-100 nm cationic particles. By simply mixing styrene and **CPiP** at various weight percent values (1, 2.5, 5, 10 and 20% of **CPiP**) and using a thermally activated radical initiator (V-50), we were able to obtain particles ranging from 30 to 90 nm (as characterized by dynamic light scattering Supplementary Fig. 2). Higher loadings of **CPiP** compared with styrene resulted in smaller, albeit more disperse, particles. Figure 3 shows the scanning electron microscope image of nanoparticles made from 5% **CPiP**/95% styrene. The average diameter obtained by dynamic light scattering was found to be 50 nm. Furthermore, the particles from stable dipersions, since the zeta potential of the 5% **CPiP** nanoparticles was found to remain above 30 mV over the range of >10 pH units (Supplementary Fig. 2). As a control, particles synthesized with styrene only (without any surfactants or **CPR** monomers) were much larger and exhibited a bimodal size distribution (Supplementary Fig. 2). These data demonstrate that the **CPiP** monomer effectively stabilises oil-in-water droplets, and that the charge is present on the particle surface. A more detailed study of this behaviour will follow, including the incorporation of other **CPR** monomers into cationic nanoparticles. In general, the ability to make charged nanoparticles in a surfactant-free, large-scale process could have far-reaching potential towards interfacial additives and biological applications^46^,^47^.

While the **CPR** monomers were successfully copolymerized to make statistical polymers and nanoparticles, we sought to augment our library of **CPR**-based macromolecules with block copolyelectrolytes (BCPEs). We synthesized block copolymers **PS-*****b*****-PCPR(CP mol%)** by growing styrene onto **PCPR** macro-chain transfer agents (macro-CTA). By varying the DP of the polystyrene block, we effectively controlled the different mole fractions of the CP functional block. We note that block copolymers **PS-*****b*****-PCPR** could also be obtained by the reverse process of growing the functional monomer **CPR** onto polystyrene macro-CTAs.

### Morphology of BCPEs

After synthesizing block copolymers of various compositions, we characterized the morphology of bulk films comprising various **CPR**-building blocks. Recent studies suggest that nanostructured BCPEs have broad implications in materials chemistry, specifically for fuel cells and batteries, if they undergo microphase segregation. For example, Winey and Elabd recently reported that BCPEs with a lamellar morphology conduct ions more effectively than cationic homopolymers, as water and ions confined within nanochannels may accelerate transport^48^. Computational studies from Olvera de la Cruz and coworkers suggest this effect may be enhanced if the conducting path is a continuous, percolating structure^17^; a microphase segregated morphology of charged and neutral blocks observed in ion-containing block copolymers^49^. With this in mind, we characterized block copolymer samples by small-angle X-ray scattering (SAXS) and TEM to understand microphase segregation in **PCPR**-containing BCPEs.

**Figure 4:**
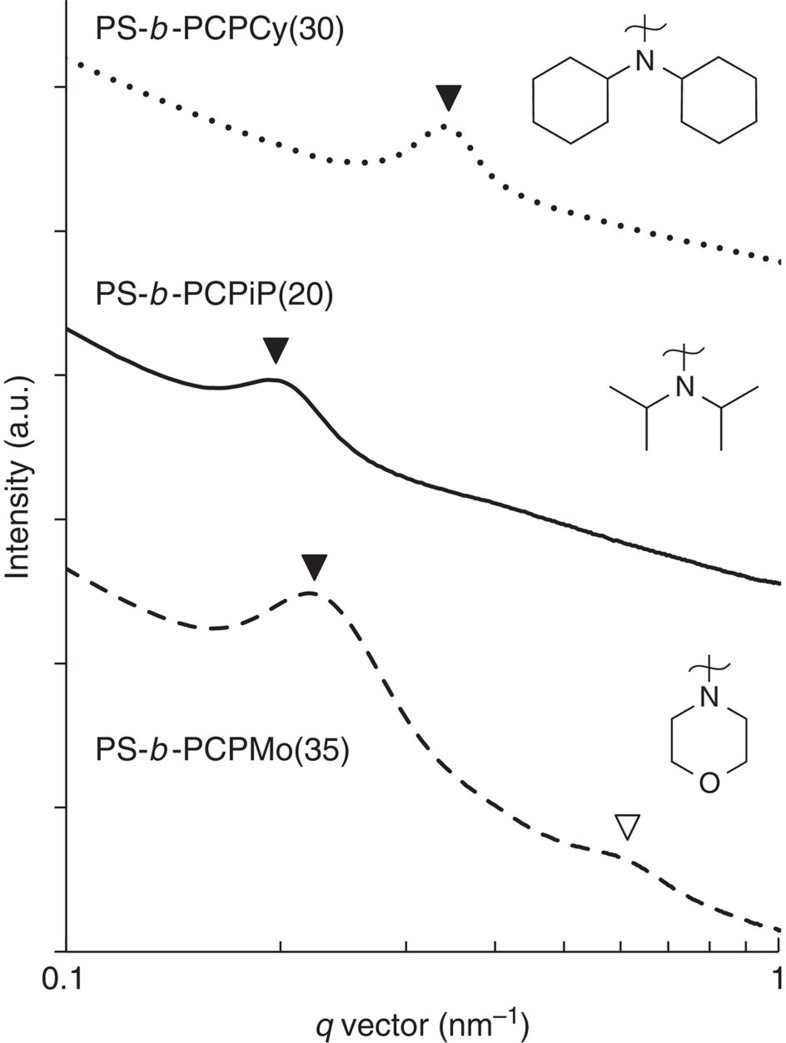
SAXS profiles of microphase separated diblock copolymers collected at 25 °C. Scattering intensity is plotted as a function of the magnitude of the scattering vector, ***q***. Filled triangles represent the primary scattering peaks and the open triangles represent the higher-order scattering peaks.

In Fig. 4, we show SAXS^50^ profiles of three representative diblock copolymers. The primary scattering peaks seen in each sample (indicated by filled triangles) is attributed to microphase separation. The scattering profile of PS-*b*-PCPMo(35) contains a higher-order peak at ***q***=3q*. This suggests the presence of a symmetric lamellar phase. The scattering profiles of the other polymers contain only one peak, which indicates a lack of long-range order. The domain spacing, *d*, of the microphase separated diblock copolymers is calculated by the equation *d*=2*π*/q*. The domain spacing values corresponding to each diblock copolymer are given in Table 1. As expected, *d*-spacing increases with molecular mass and molar fraction of styrene. We next sought to probe the potential application of CP-based polyelectrolytes in electrochemical devices^11^,^17^.

**Figure 5:**
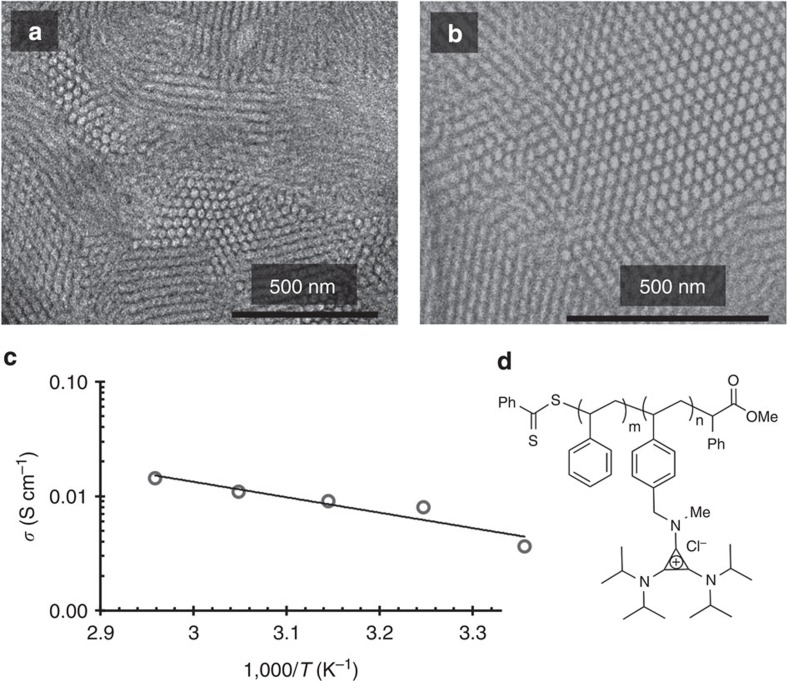
Morphology and ionic conductivity of bulk PS-*b*-PCPiP films. (**a** and **b**) Two representative TEM images of **PS-*****b*****-PCPiP(20)** reveal a morphology of hexagonally packed cylinders (*d*-spacing=29 nm; the light colour corresponds to PS). (**c**) Ionic conductivity as a function of inverse temperature, from 25 to 65 °C, for (**d**) **PS-*****b*****-PCPiP(20)** (ion-exchange capacity, IEC=1.3, at 90% RH).

Ion conductivity experiments were performed on **PS-*****b*****-PCPiP(20)** using electrochemical impedance spectroscopy. As conductivity is closely related to morphology, we complemented our SAXS experiments with transmission electron microscopy (TEM). A **PS-*****b*****-PCPiP(20)** sample (drop cast, no annealing) was microtomed and imaged by TEM (Fig. 5a,b). Even without staining, we clearly observe microphase segregation (cylindrical morphology) (Supplementary Fig. 4). Staining with RuO_4_ vapour for 2 min preferentially stains the cationic block and helps to visualize the internal structure. The electron micrographs obtained (Fig. 5a,b) show hexagonally packed cylinders in different orientations. The domain spacing by TEM was 29 nm, which is consistent with the domain spacing determined by SAXS (31 nm; see Table 1). The lighter colour of the cylinders with respect to the matrix in Fig. 5a,b indicates that PS cylinders are embedded in a **PCPiP** matrix. The stained **PCPiP** block scatters more electrons, and therefore appears darker by TEM (see Supplementary Fig. 4). The non-functionalised PS cylinders in Fig. 5a,b occupy a very large fraction of the image because the mole fraction of the functional block in this polymer is only 20%. The continuous nature of the conducting phase matrix observed in charged diblock copolymers is expected to facilitate ion transport^49^, and is consistent with the ideal percolating structure found by computational modelling^17^.

## Discussion

The in-plane conductivity, *σ*, of **PS-*****b*****-PCPiP(20)** equilibrated in humid air with 90% relative humidity (RH) was measured as a function of increasing temperature from 25 to 65 °C (Fig. 5c). To ensure equilibration, samples were initially annealed for 1 week at 90% RH at 25 °C and for 48 h at each subsequent temperature of interest. The straight line in Fig. 5c is the least-squares fit through the equilibrated conductivity data at each temperature value. In principle, the change in conductivity with temperature, shown in Fig. 5c, could either be due to changes in the mobility of chloride ions or to a change in ion concentration in the membrane. The fact that ion concentration in the membranes is constant indicates that the slope of the line provides an estimate of the activation energy for transport of chloride ions through the membrane (Arrhenius law). The estimated activation energy for this system is 25 kJ mol^−1^. This value is comparable to that reported previously for the imidazolium-containing diblock copolymer analogue in water, poly(styrene-*b*-4-vinylbenzyltrimethylimidazolium chloride) (PS-*b*-PIm(35)), 27 kJ mol^−1^ (ref. 43). However, at room temperature, the conductivity of the PS-*b*-PCPiP(20) polymer (ion-exchange capacity, IEC=1.3 meq g^−1^) is rather high, 0.004 S cm^−1^, considering the low water uptake, *λ*_w_=7, of this membrane (*λ*_w_ is the number of water molecules per chloride ion in the membrane). This value of *λ*_w_ is four times lower than the value obtained for PS-*b*-PIm(35) immersed in water, for the same conductivity (*λ*_w_=30, *σ*=0.004 S cm^−1^), and higher IEC (2.1 meq g^−1^)^43^. These results indicate that the CP-based polyelectrolytes conduct ions more effectively than the optimized membranes from imidazolium-containing polymers, with a minimum amount of water present. Further tuning of the functional groups, backbone structure and morphology is expected to result in polyelectrolytes with exceptionally high ion conductivities^51^,^52^,^53^,^54^.

In conclusion, we have introduced a new family of electron-rich cationic polyelectrolytes based on the CP ion building block. The robust, efficient and orthogonal chemistry to synthesise the monomers provides facile access to a variety of polymers by RAFT and to well-defined cationic nanoparticles by surfactant-free emulsion polymerization. The nanoparticles exhibit high charge density on the surface and stability over a wide range of pH values. The family of linear polymers is characterized by widely variable physical properties, which are highly dependent on the amino substituents flanking the aromatic cation. Through TEM and SAXS measurements, we observed microphase segregation in bulk samples of the BCPE. In diblock copolymers, the domain spacing increased with increasing styrene content (the length of the functional block was fixed). Compared with imidazolium analogues, CP-based BCPEss show superior properties as ion conductive materials, and we anticipate that further optimization will lead to improved performance. Future studies will be aimed at probing the structure–property relationships of these materials by expanding our **PCPR** library through adjustment of the polymer backbone, modular functional groups, block copolymer composition and CP counterion. Moreover, other **CPR** monomers will be incorporated into nanoparticles via the one-pot emulsion polymerization to assess their efficacy in various biomedical applications and membrane technologies. With such modularity, this new class of CP-based polyelectrolytes offers a wealth of functionality that translates to significant potential across a broad array of applications.

## Methods

### Synthesis of *N*-methyl-1-(2,3-bis(dicyclohexylamino)cyclopropenium)-4-vinylbenzylamine chloride (CPCy)

To a dry round bottom flask of 2,3-bis(dicyclohexylamino)-1-chlorocyclopropenium chloride (22.1 g, 47.3 mmol, 1.0 equiv) under argon was added CH_2_Cl_2_ (150 ml) and triethylamine (6.54 ml, 47.3 mmol, 1.0 equiv). *N*-Methyl-4-vinylbenzylamine (7.74 g, 47.3 mmol and 1.0 equiv) was then slowly added to the solution and the reaction was stirred overnight. CH_2_Cl_2_ (700 ml) was added and the mixture was washed with 1 M HCl (3 × 200 ml) and brine (1 × 200 ml), dried with anhydrous sodium sulfate and concentrated *in vacuo* to yield a crude solid. The crude product was purified with silica gel chromatography (EtOAc then 5% iPrOH in CH_2_Cl_2_) to yield an off-white solid (23.5 g, 40.6 mmol, 86% yield). Monomers were characterized by ^1^H and ^13^C NMR (see Supplementary Figs 5–10), and all new compounds were characterized by NMR and mass spectrometry (see Supplementary Notes and Methods).

### Sample synthesis of PCPR: synthesis of PCPCy

To a dry 20 ml (see Supplementary Notes and Methods for details) scintillation vial, **CPCy** (6.0 g, 10.4 mmol, 60.0 equiv), methyl 2-(phenylcarbonothioylthio)-2-phenylacetate (52.3 mg, 1.73 mmol, 1.0 equiv), AIBN (4.3 mg, 0.26 mmol, 0.15 equiv) and DMF (6.0 ml) were added and vortexed to form a homogenous solution. This solution was transferred to a flame-dried ampule. After 4 freeze-pump-thaw cycles, the ampule was sealed under vacuum. The polymerization was run for 12 h at 80 °C with vigorous stirring. The reaction mixture was precipitated from CH_2_Cl_2_ into 1,4-dioxane three times to remove monomer. The polymer was then precipitated an additional three times into hexanes to remove residual 1,4-dioxane. Drying *in vacuo* yielded the pure polymer as a pink powder (5.3 g, 88% yield). Homopolymers were characterized by ^1^H NMR (see Supplementary Figs 11–13).

### Small-angle X-ray scattering

Thick polymer samples (1 mm) were prepared by pressing the powder into a teflon washer. Synchrotron SAXS measurements were performed using the 7.3.3 beamline at the Advanced Light Source (ALS, Lawrence Berkeley National Laboratory). The wavelength *λ* of the incident X-ray beam was 0.124 nm (Δ*λ*/*λ*=10^–4^) and a sample-to-detector distance of 4 m. The resulting two-dimensional scattering data were averaged azimuthally to obtain intensity versus magnitude of the scattering wave vector ***q*** (***q***=4*π* sin(*θ*/2)/*λ*, where *θ* is the scattering angle). All of the scattering profiles were azimuthally symmetric. The scattering data were corrected for the detector dark current and the scattering from air and Kapton windows.

### Electrochemical impedance spectroscopy

 In-plane chloride conductivity of hydrated membranes with dimensions 2 cm × 1 cm × 450 μm was measured by AC impedance spectroscopy using platinum electrodes in the standard four probe configuration using a BekkTech sample clamp. Polymer films of PS-b-PCPiP(20) were prepared by drop casting a 100 mg ml^−1^ solution of polymer onto a clean Teflon substrate. In-plane chloride conductivity of a hydrated membrane composed of **PS-*****b*****-PCPiP(20)** (calculated molecular mass=31 KDa, DP=174) with dimensions 2 cm × 1 cm × 450 μm was measured by AC impedance spectroscopy using platinum electrodes in the standard four probe configuration using a BekkTech sample clamp. Conductivities were collected under humidified conditions, and temperature and RH were controlled by an environmental chamber (Qualitest). Data were collected using 10 mV amplitude over a frequency range of 1 Hz–10 MHz. Separate experiments were conducted to ensure that the response of the sample was linear in this window. Samples were annealed at the temperature of interest for 24–48 h until the measured impedance did not change. Conductivity, *σ*, is given by equation (1):


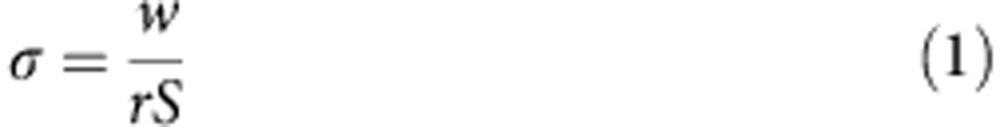


where *S* is the cross-sectional area of sample film, *r* is the intercept of the Nyquist semi-circle on the real axis (Ω) and *w* is the distance between the inner platinum electrodes.

### Transmission electron microscopy

Films of **PS-*****b*****-PCPiP(20)** (calculated molecular mass=33.4 KDa, DP=200) were prepared by drop casting a 100 mg ml^−1^ solution of polymer onto a clean Teflon substrate. After allowing to dry for 24 h, the film was sectioned with Leica UltraCut 6 ultramicrotome at –40 °C, nominal thickness 70 nm using a Diatome Cryo 35° diamond knife. Sections were placed on 300 mesh copper grids with homemade lacey carbon film on top. The sections were stained with RuO4 vapour for 2 min, which preferentially stained the **PCPiP** block. Sections were imaged with FEI Tecnai F20 TEM operated at 200 kV. Images were analysed using ImageJ 1.48v software.

## Author contributions

All authors contributed to the conception and execution of experiments. Materials were synthesized by J.L.F., Y.J., S.D.B. and K.L.K. Ionic conductivity measurements and X-ray scattering were performed by P.C. All authors contributed to data analysis. The paper was written by J.L.F., Y.J. and L.C., with input from all authors.

## Additional information

**Accession codes:** A patent related to this research has been issued, ‘Cyclopropenium Polymers and Methods for Making the Same,’ WO 2014/022365 A1.

**How to cite this article:** Jiang, Y. *et al.* The evolution of cyclopropenium ions into functional polyelectrolytes. *Nat. Commun.* 6:5950 doi: 10.1038/ncomms6950 (2015).

## Supplementary Material

Supplementary Information Supplementary Figures 1-13, Supplementary Table 1-2, Supplementary Note 1, Supplementary Methods and Supplementary Reference

## Figures and Tables

**Table 1:** Characterization of PS-*b*-PCPR (CP mol%) block copolymers.

**Sample Name**	**MM/kg mol**^−**1**^	**CP% by DP**	**CP% by MM**	**SAXS domain spacing (nm)**
**PS-*****b*****-PCPCy(45)**	40	45%	80%	15
**PS-*****b*****-PCPCy(30)**	50	30%	70%	18
**PS-*****b*****-PCPiP(50)**	20	50%	80%	18
**PS-*****b*****-PCPiP(30)**	27	30%	60%	24
**PS-*****b*****-PCPiP(20)**	30	20%	50%	31
**PS-*****b*****-PCPMo(50)**	38	50%	80%	26
**PS-*****b*****-PCPMo(35)**	42	35%	70%	28

CP, cyclopropenium; DP, degree of polymerization; NMR, nuclear magnetic resonance; SAXS, small-angle X-ray scattering

Block copolymers were synthesized by the addition of styrene to the three homopolymers, and domain spacing was calculated by SAXS (Supplementary Fig. 3). Molecular mass (MM) was determined by ^1^H NMR spectroscopy.

## References

[b1] Hawker C. J. & Wooley K. L. The convergence of synthetic organic and polymer chemistries. Science 309, 1200–1205 (2005). 16109874 10.1126/science.1109778

[b2] Hunt J. N. *et al.* Tunable, High modulus hydrogels driven by ionic coacervation. Adv. Mater. 23, 2327–2331 (2011). 21491513 10.1002/adma.201004230

[b3] Leibfarth F. A. *et al.* A facile route to ketene-functionalized polymers for general materials applications. Nat. Chem. 2, 207–212 (2010). 21124478 10.1038/nchem.538

[b4] Lodge T. P. A unique platform for materials design. Science 321, 50–51 (2008). 18599764 10.1126/science.1159652

[b5] Gao R., Wang D., Heflin J. R. & Long T. E. Imidazolium sulfonate-containing pentablock copolymer-ionic liquid membranes for electroactive actuators. J. Mater. Chem. 22, 13473–13476 (2012).

[b6] Hallinan D. T. & Balsara N. P. Polymer electrolytes. Annu. Rev. Mater. Res. 43, 503–525 (2013).

[b7] De Smedt S., Demeester J. & Hennink W. Cationic polymer based gene delivery systems. Pharm. Res. 17, 113–126 (2000). 10751024 10.1023/a:1007548826495

[b8] Samal S. K. *et al.* Cationic polymers and their therapeutic potential. Chem. Soc. Rev. 41, 7147–7194 (2012). 22885409 10.1039/c2cs35094g

[b9] Pan J., Chen C., Zhuang L. & Lu J. Designing advanced alkaline polymer electrolytes for fuel cell applications. Acc. Chem. Res. 45, 473–481 (2011). 22075175 10.1021/ar200201x

[b10] Hickner M. A., Herring A. M. & Coughlin E. B. Anion exchange membranes: current status and moving forward. J. Polym. Sci. B Polym. Phys. 51, 1727–1735 (2013).

[b11] Chen Y. *et al.* Enhancement of anhydrous proton transport by supramolecular nanochannels in comb polymers. Nat. Chem. 2, 503–508 (2010). 20489721 10.1038/nchem.629

[b12] Elimelech M. & Phillip W. A. The future of seawater desalination: energy, technology, and the environment. Science 333, 712–717 (2011). 21817042 10.1126/science.1200488

[b13] Gin D. L. & Noble R. D. Designing the next generation of chemical separation membranes. Science 332, 674–676 (2011). 21551053 10.1126/science.1203771

[b14] Jangu C. & Long T. E. Phosphonium cation-containing polymers: from ionic liquids to polyelectrolytes. Polymer 55, 3298–3304 (2014).

[b15] Qiu B., Lin B., Qiu L. & Yan F. Alkaline imidazolium- and quaternary ammonium-functionalized anion exchange membranes for alkaline fuel cell applications. J. Mater. Chem. 22, 1040–1045 (2012).

[b16] Yuan J., Mecerreyes D. & Antonietti M. Poly(ionic liquid)s: an update. Prog. Polym. Sci. 38, 1009–1036 (2013).

[b17] Sing C. E., Zwanikken J. W. & Olvera de la Cruz M. Electrostatic control of block copolymer morphology. Nat. Mater. 13, 694–698 (2014). 24907928 10.1038/nmat4001

[b18] Steele B. C. H. & Heinzel A. Materials for fuel-cell technologies. Nature 414, 345–352 (2001). 11713541 10.1038/35104620

[b19] Curnow O. J., MacFarlane D. R. & Walst K. J. Triaminocyclopropenium salts as ionic liquids. Chem. Commun. 47, 10248–10250 (2011). 10.1039/c1cc13979g 21847498

[b20] Hückel E. Grundzüge der Theorie ungesättiger und aromatischer Verbindungen. Z. Physik 70, 77–85 (1938).

[b21] Breslow R. Synthesis of the s-triphenylcyclopropenyl cation. J. Am. Chem. Soc. 79, 5318–5318 (1957).

[b22] Bandar J. S. & Lambert T. H. Aminocyclopropenium ions: synthesis, properties, and applications. Synthesis 45, 2485–2498 (2013).

[b23] Yoshida Z. & Tawara Y. Aminocyclopropenium ion. J. Am. Chem. Soc. 93, 2573–2574 (1971).

[b24] Yoshida Z.-I., Tawara Y., Hirota S. & Ogoshi H. Vibrational spectrum of trisdimethylaminocyclopropenium perchlorate. Bull. Chem. Soc. Jpn 47, 797–800 (1974).

[b25] Kerber R. C. & Hsu C.-M. Substituent effects on cyclopropenium ions. J. Am. Chem. Soc. 95, 3239–3245 (1973).

[b26] Huddleston J. G. *et al.* Characterization and comparison of hydrophilic and hydrophobic room temperature ionic liquids incorporating the imidazolium cation. Green Chem. 3, 156–164 (2001).

[b27] Bruns H. *et al.* Synthesis and coordination properties of nitrogen(i)-based ligands. Angew. Chem. Int. Ed. 49, 3680–3683 (2010). 10.1002/anie.200906168 20391546

[b28] Bandar J. S. & Lambert T. H. Enantioselective brønsted base catalysis with chiral cyclopropenimines. J. Am. Chem. Soc. 134, 5552–5555 (2012). 22417078 10.1021/ja3015764

[b29] Bandar J. S. & Lambert T. H. Cyclopropenimine-catalyzed enantioselective mannich reactions of tert-butyl glycinates with n-boc-imines. J. Am. Chem. Soc. 135, 11799–11802 (2013). 23906087 10.1021/ja407277a PMC3804837

[b30] Wilde M. M. D. & Gravel M. Bis(amino)cyclopropenylidenes as organocatalysts for acyl anion and extended umpolung reactions. Angew. Chem. Int. Ed. 52, 12651–12654 (2013). 10.1002/anie.201307167 24123480

[b31] Weidner C. H. & Long T. E. Synthesis and characterization of 3-aryl-2-(polystyryl)cyclopropenones via cyclopropenium ion substitution on polystyrene. J. Polym. Chem. A Polym. Chem. 33, 1–6 (1995).

[b32] Hawker C. J., Bosman A. W. & Harth E. New polymer synthesis by nitroxide mediated living radical polymerizations. Chem. Rev. 101, 3661–3688 (2001). 11740918 10.1021/cr990119u

[b33] Matyjaszewski K. & Xia J. Atom transfer radical polymerization. Chem. Rev. 101, 2921–2990 (2001). 11749397 10.1021/cr940534g

[b34] Chiefari J. *et al.* Living free-radical polymerization by reversible addition-fragmentation chain transfer: the RAFT process. Macromolecules 31, 5559–5562 (1998).

[b35] Peart P. A. & Tovar J. D. Poly(cyclopropenone)s: formal inclusion of the smallest Hückel aromatic into π-conjugated polymers. J. Org. Chem. 75, 5689–5696 (2010). 20704438 10.1021/jo101108f

[b36] Curnow O. J., Holmes M. T., Ratten L. C., Walst K. J. & Yunis R. A facile route to functionalised, protic and chiral ionic liquids based on the triaminocyclopropenium cation. RSC Adv. 2, 10794–10797 (2012).

[b37] Kolb H. C., Finn M. G. & Sharpless K. B. Click chemistry: diverse chemical function from a few good reactions. Angew. Chem., Int. Ed. 40, 2004–2021 (2001). 10.1002/1521-3773(20010601)40:11<2004::AID-ANIE2004>3.0.CO;2-5 11433435

[b38] Tobey S. W. & West R. Pentachlorocyclopropane. J. Am. Chem. Soc. 88, 2478–2481 (1966).

[b39] Hemp S. T. *et al.* Comparing ammonium and phosphonium polymerized ionic liquids: thermal analysis, conductivity, and morphology. Macromol. Chem. Phys. 214, 2099–2107 (2013).

[b40] Yuan J. & Antonietti M. Poly(ionic liquid)s: polymers expanding classical property profiles. Polymer 52, 1469–1482 (2011).

[b41] Wang R. & Lowe A. B. RAFT polymerization of styrenic-based phosphonium monomers and a new family of well-defined statistical and block polyampholytes. J. Polym. Sci. A Polym. Chem. 45, 2468–2483 (2007).

[b42] Texter J. Anion responsive imidazolium-based polymers. Macromol. Rapid Commun. 33, 1996–2014 (2012). 22997012 10.1002/marc.201200525

[b43] Sudre G., Inceoglu S., Cotanda P. & Balsara N. P. Influence of bound ion on the morphology and conductivity of anion-conducting block copolymers. Macromolecules 46, 1519–1527 (2013).

[b44] Weber R. L. *et al.* Thermal and ion transport properties of hydrophilic and hydrophobic polymerized styrenic imidazolium ionic liquids. J. Polym. Sci. B Polym. Phys 49, 1287–1296 (2011).

[b45] Ramos J., Forcada J. & Hidalgo-Alvarez R. Cationic polymer nanoparticles and nanogels: from synthesis to biotechnological applications. Chem. Rev. 114, 367–428 (2013). 24003911 10.1021/cr3002643

[b46] Rothberg J. M. *et al.* An integrated semiconductor device enabling non-optical genome sequencing. Nature 475, 348–352 (2011). 21776081 10.1038/nature10242

[b47] Nederberg F. *et al.* Biodegradable nanostructures with selective lysis of microbial membranes. Nat. Chem. 3, 409–414 (2011). 21505501 10.1038/nchem.1012

[b48] Ye Y., Sharick S., Davis E. M., Winey K. I. & Elabd Y. A. High hydroxide conductivity in polymerized ionic liquid block copolymers. ACS Macro Lett. 2, 575–580 (2013). 10.1021/mz400210a 35581784

[b49] Park M. J. *et al.* Increased water retention in polymer electrolyte membranes at elevated temperatures assisted by capillary condensation. Nano Lett. 7, 3547–3552 (2007). 17960948 10.1021/nl072617l

[b50] Alexander H. *et al.* A SAXS/WAXS/GISAXS beamline with multilayer monochromator. J. Phys. Conf. Ser 247, 012007 (2010).

[b51] Schmidt-Rohr K. & Chen Q. Parallel cylindrical water nanochannels in Nafion fuel-cell membranes. Nat. Mater. 7, 75–83 (2008). 18066069 10.1038/nmat2074

[b52] Choi J.-H., Ye Y., Elabd Y. A. & Winey K. I. Network structure and strong microphase separation for high ion conductivity in polymerized ionic liquid block copolymers. Macromolecules 46, 5290–5300 (2013).

[b53] Hoarfrost M. L., Tyagi M. S., Segalman R. A. & Reimer J. A. Effect of confinement on proton transport mechanisms in block copolymer/ionic liquid membranes. Macromolecules 45, 3112–3120 (2012).

[b54] Ye Y., Choi J.-H., Winey K. I. & Elabd Y. A. Polymerized ionic liquid block and random copolymers: effect of weak microphase separation on ion transport. Macromolecules 45, 7027–7035 (2012).

