# Supplementary material for: The evolution of cyclopropenium ions into functional polyelectrolytes
Source: Nat Commun. 2015 Jan 9;6:5950. doi: 10.1038/ncomms6950 (PMC4354017; doi:10.1038/ncomms6950)
Supplement: Supplementary Information — Supplementary Figures 1-13, Supplementary Table 1-2, Supplementary Note 1, Supplementary Methods and Supplementary Reference [file ncomms6950-s1.pdf]

Supplementary Information

Supplementary Figures.

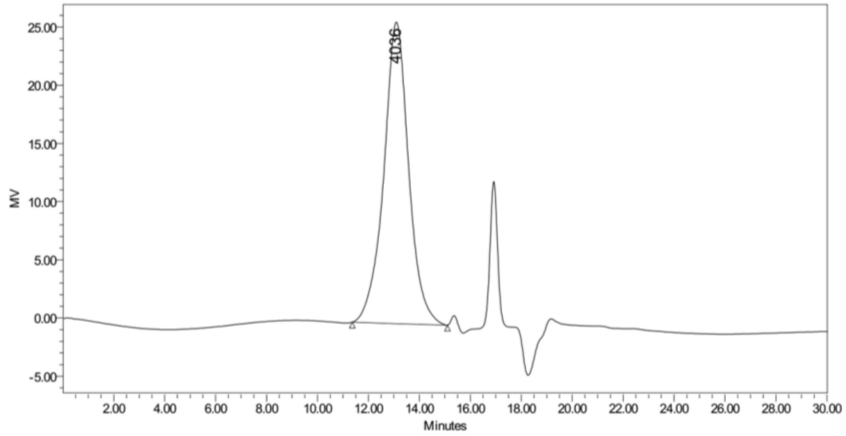

Supplementary Figure 1. Size exclusion chromatography **(**SEC) trace of **PCPMo**. The dispersity ($Đ$) was found to be 1.3 (see Supplementary Table 2).

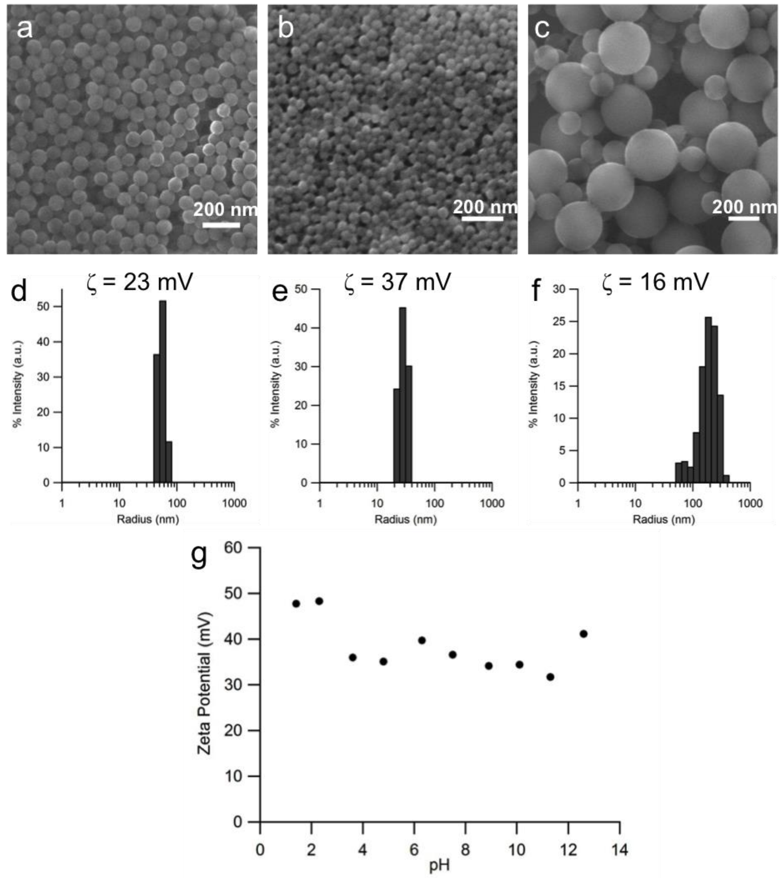

Supplementary Figure 2. Particles synthesized by surfactant-free emulsion polymerization. scanning electron microscopy (SEM) images and dynamic light scattering (DLS) histograms, respectively, for **1 wt.% CPiP** (a, d), **5 wt.% CPiP** (b, e) and **0 wt.% CPiP** (PS only) (c, f). Plot of zeta potential at a range of pH values for **5 wt.% CPiP**.

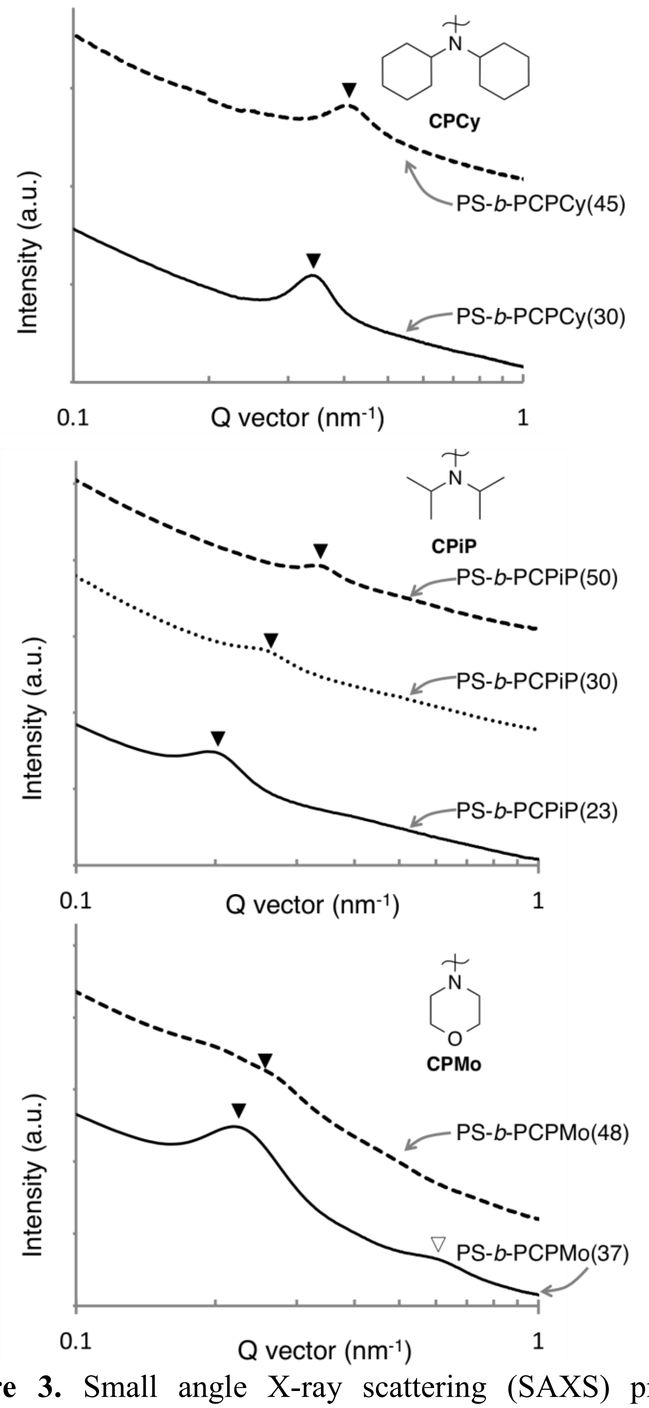

Supplementary Figure 3. Small angle X-ray scattering (SAXS) profiles of microphase segregated diblock copolymers collected at 25 °C. Scattering intensity is plotted as a function of the magnitude of the scattering vector, *q*. Filled triangles represent the primary scattering peaks, and the open triangles represent the higher order scattering peaks.

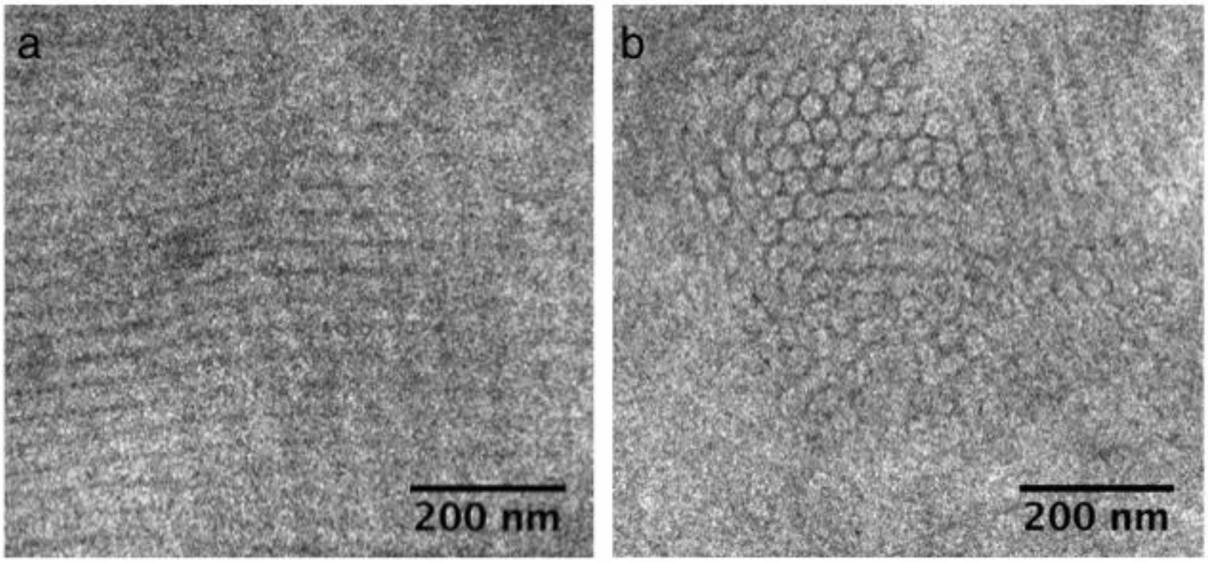

Supplementary Figure 4. Transmission electron microscopy **(**TEM) images of **PS-*b*-PCPiP(20)** without exposure to RuO_4 vapor. **a.** Cross-section of the cylinders. **b.** Hexagonally packed cylinders orientated orthogonal to the section.

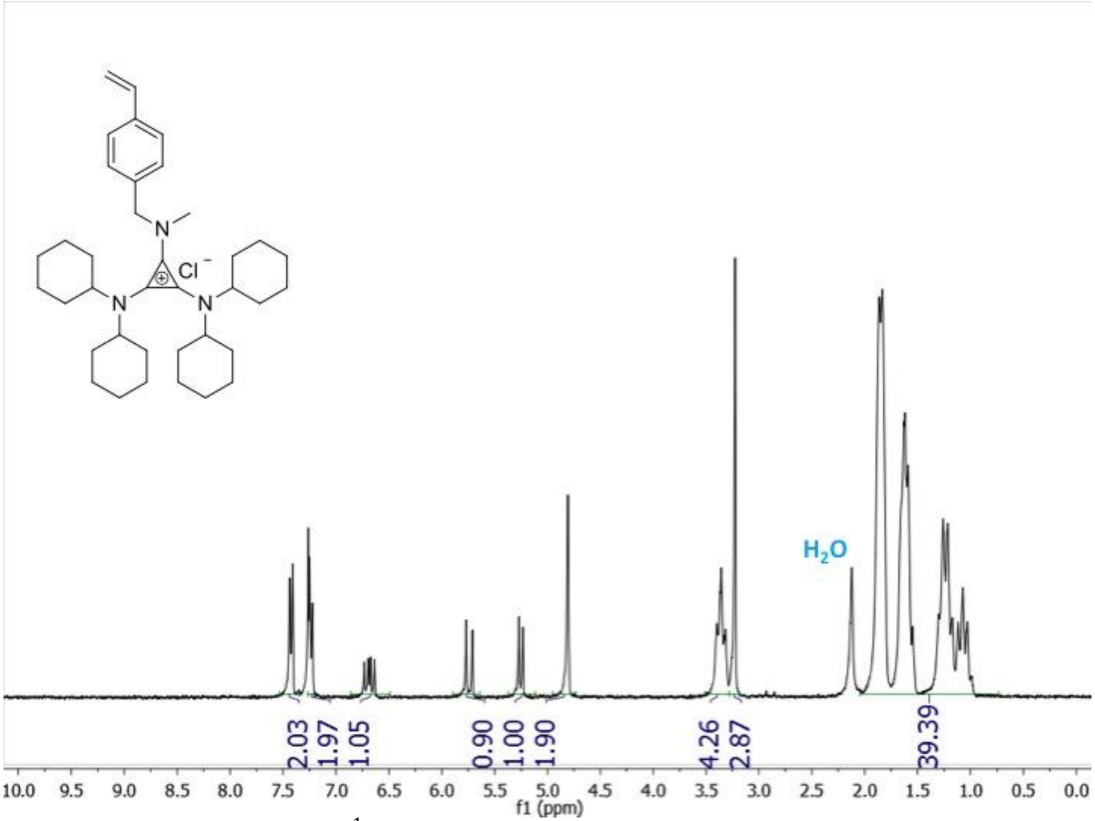

Supplementary Figure 5. ^{1}H-NMR spectrum of *N*-methyl-1-(2,3-bis(dicyclohexylamino)cyclopropenium)-4-vinylbenzylamine chloride (**CPCy**)

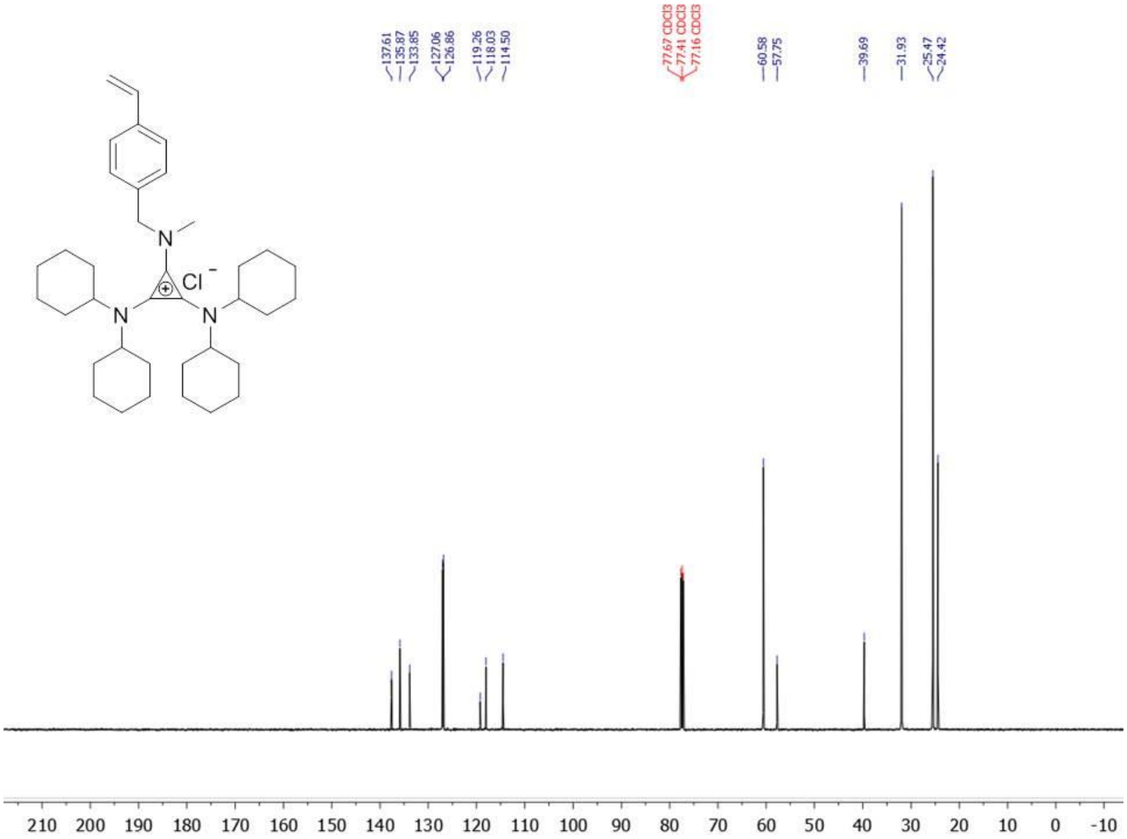

Supplementary Figure 6. ^{13}C NMR spectrum of *N*-methyl-1-(2,3-bis(dicyclohexylamino)cyclopropenium)-4-vinylbenzylamine chloride (**CPCy**)

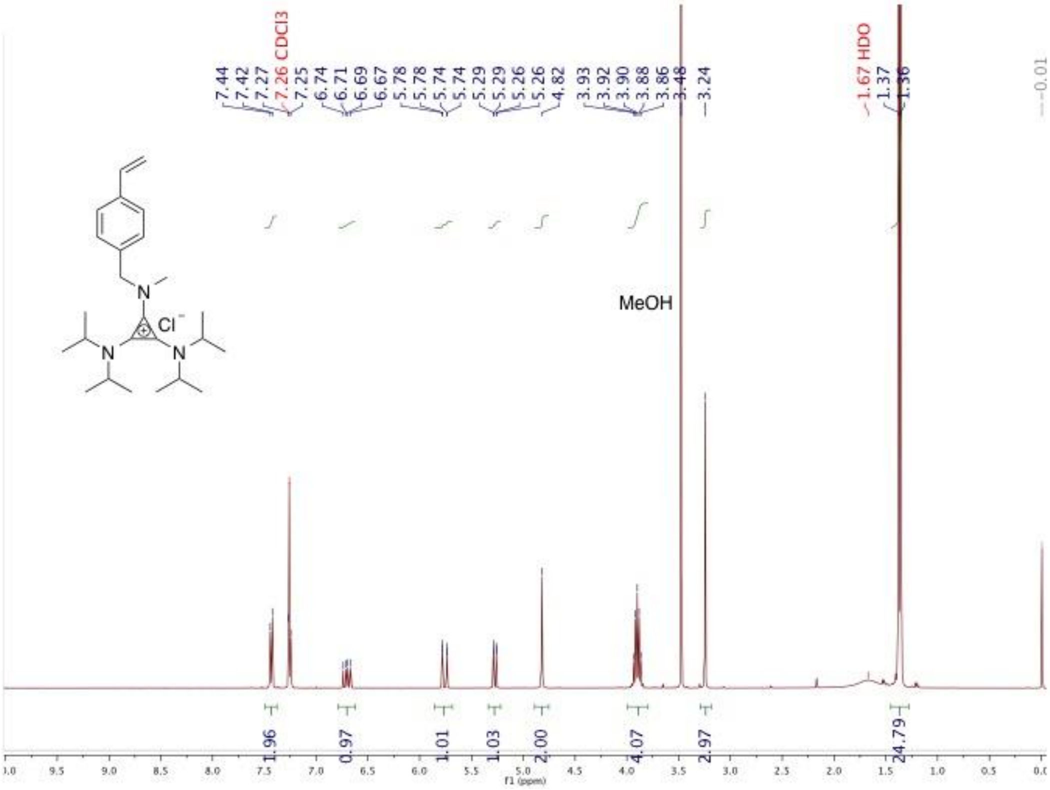

Supplementary Figure 7. ^{1}H NMR spectrum of *N*-methyl-1-(2,3-bis(diisopropylamino)cyclopropenium)-4-vinylbenzylamine chloride (**CPiP**)

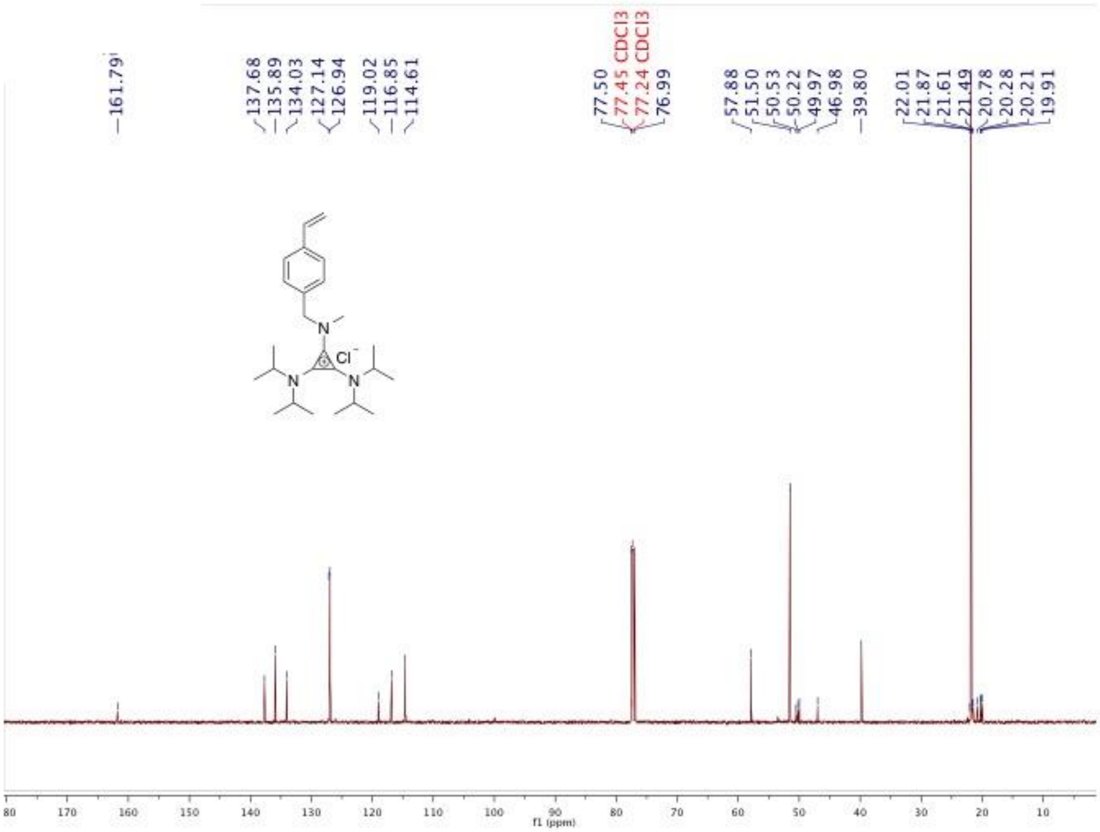

Supplementary Figure 8. ^{13}C NMR spectrum of *N*-methyl-1-(2,3-bis(diisopropylamino)cyclopropenium)-4-vinylbenzylamine chloride (**CPiP**)

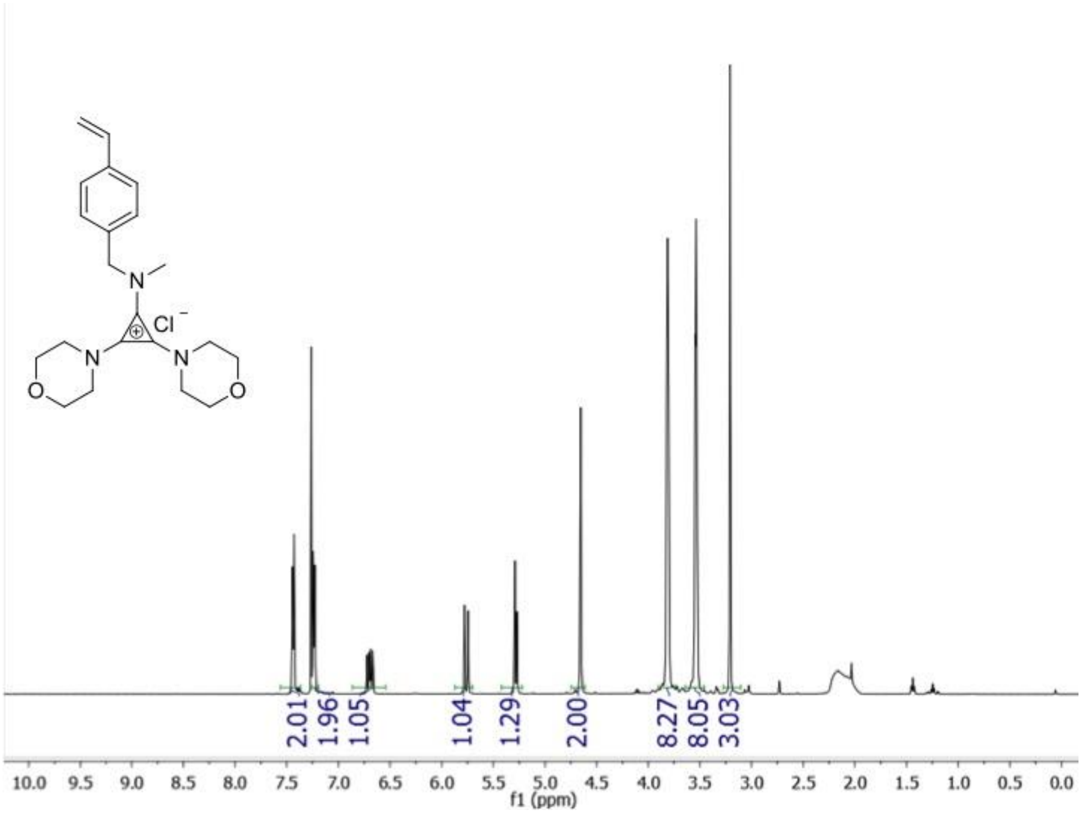

Supplementary Figure 9. ^{1}H NMR spectrum of *N*-methyl-1-(2,3-bis(morpholino)cyclopropenium)-4-vinylbenzylamine chloride (**CPMo**)

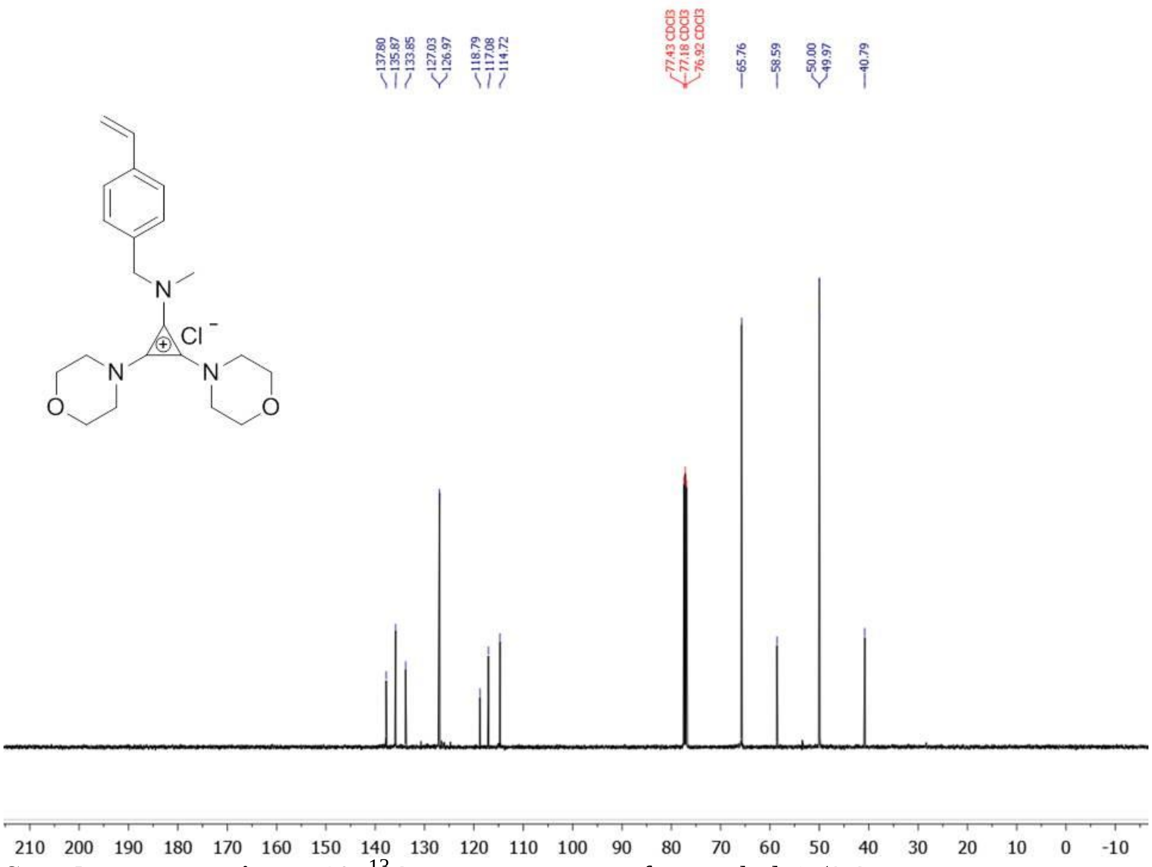

Supplementary Figure 10. ^{13}C NMR spectrum of *N*-methyl-1-(2,3-bis(morpholino)cyclopropenium)-4-vinylbenzylamine chloride (**CPMo**)

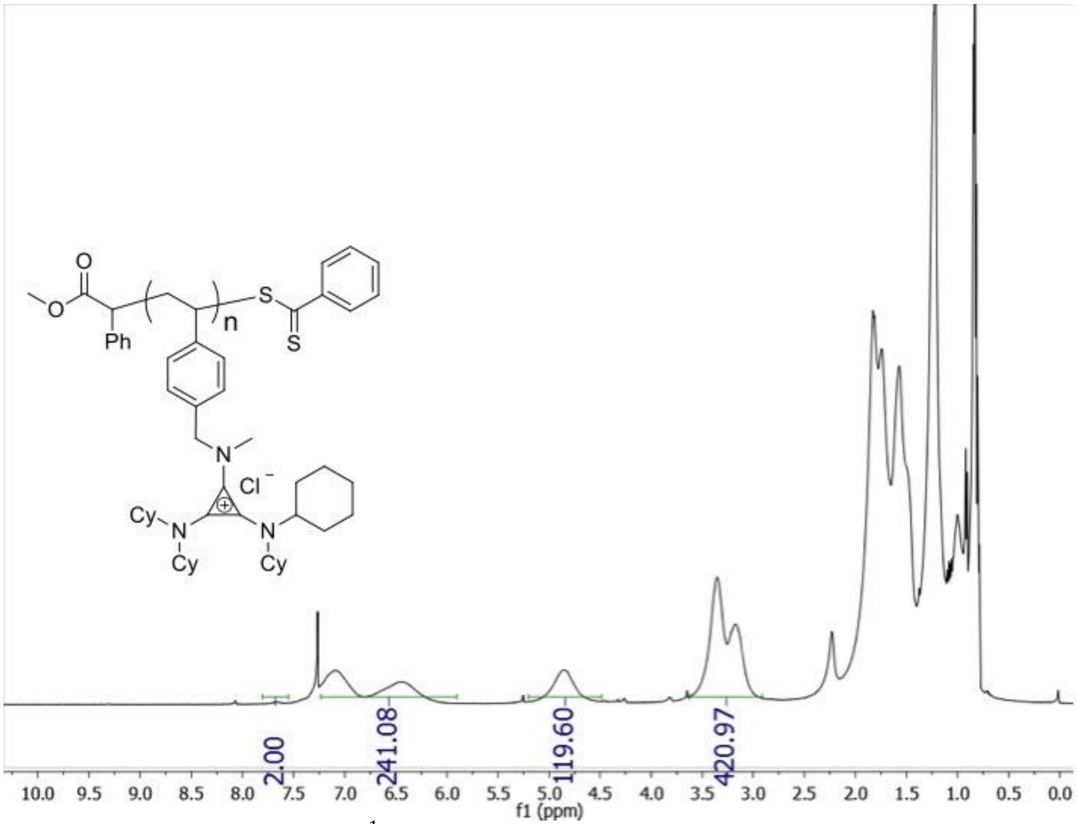

Supplementary Figure 11. ^{1}H NMR spectrum of **PCPCy**

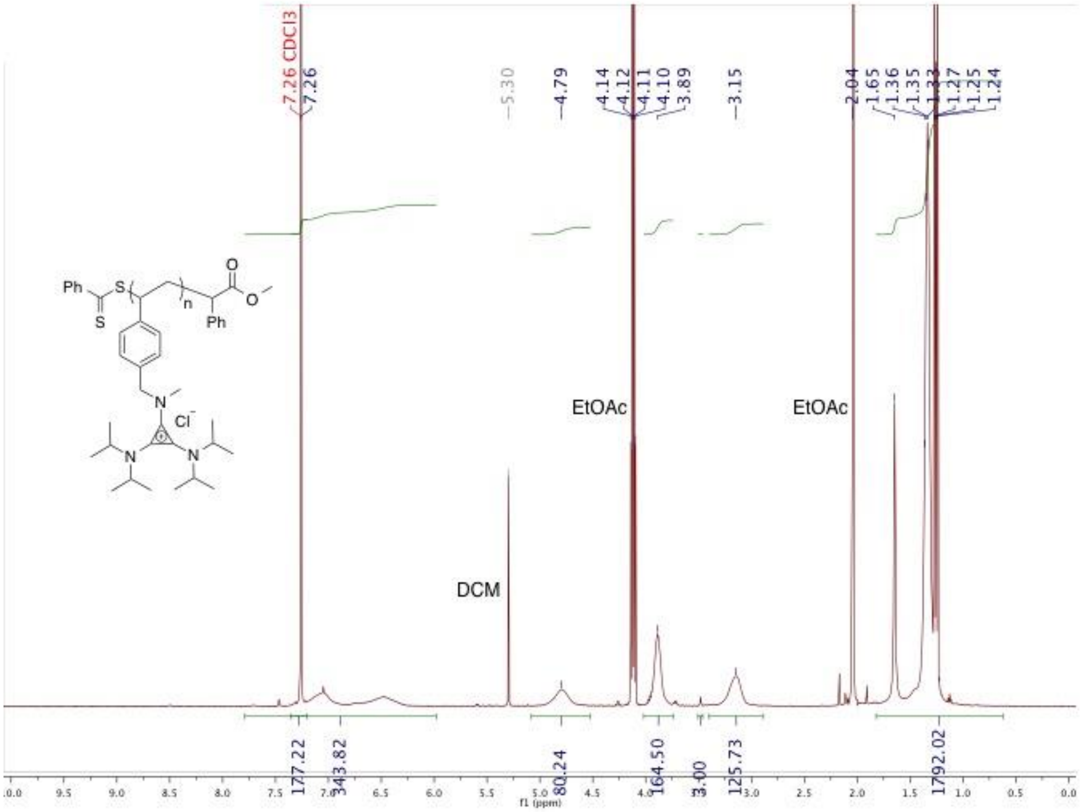

Supplementary Figure 12. ^{1}H NMR spectrum of **PCPiP**

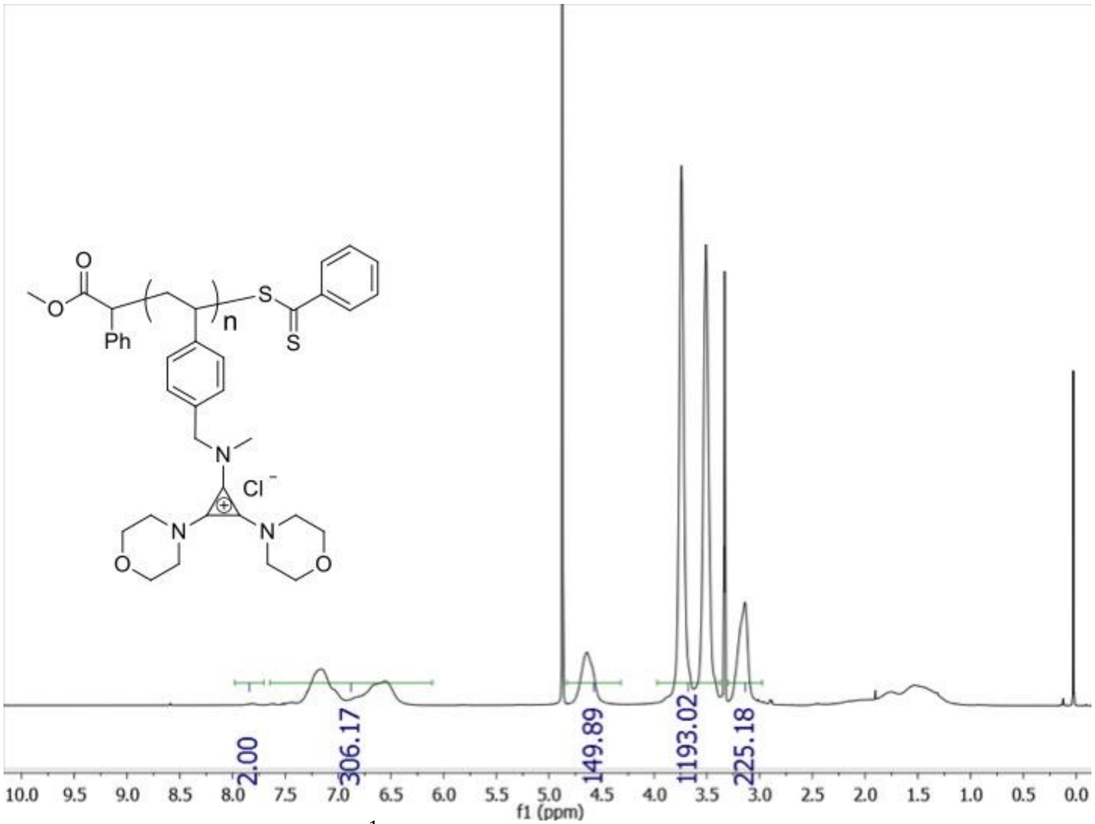

Supplementary Figure 13. ^{1}H NMR spectrum of **PCPMo**

Supplementary Tables

Supplementary Table 1: Characterization of PCPR homopolymers. As the size and hydrophobicity of the alkyl chains decreased from **PCPCy** to **PCPMo**, conversion of the monomers became noticeably lower and T_{dec} was found to increase. **PCPMo** was found to have a T_g of 160°C. Solubilities also depended on the hydrophilicity of the alkyl chains.

					**Solubilities**[b]				
Sample	MM /kg mol^{-1}	DP	T_{dec}	T_g[a]	$CHCl_3$	CH_2Cl_2	ROH[c]	H_2O	THF
**PCPCy**	35	60	160° C	N/A	□	□	□	□	□
**PCPiP**	17	40	225° C	N/A	□	□	□	□	□
**PCPMo**	29	75	310° C	160° C	□	□	□	□	□

a) Both **PCPCy** and **PCPiP** decomposed before reaching any recordable T_g. b) All homopolymers are soluble in DMF and DMSO and insoluble in hexanes, ethyl acetate, and diethyl ether. c) ROH = methanol and ethanol. Only **PCPMo** was not soluble in isopropanol. Note: □□means soluble and □ is insoluble.

Supplementary Table 2. SEC data for **PCPMo** (see Supplementary Figure 1).

MM (Daltons)	Mn (Daltons)	MP (Daltons)	Dispersity (*Đ*)	Peak Name	RT	Area	% Area	Height
4587	3422	4036	1.340446	Broad	13.094	1762186	100.00	25897

MM = mass average molecular mass. Mn = number average molecular mass. MP = molecular mass at the peak maximum. RT = retention time.

Supplementary Note 1

All materials were purchased from Sigma Aldrich and were used without further purification except as noted below. Methylene chloride (CH_2Cl_2), tetrahydrofuran (THF), and *N*,*N*-dimethylformamide (DMF) were dried using a J.C. Meyer solvent purification system. Styrene was filtered through basic alumina to remove radical inhibitor before use in polymerizations. Deuterated solvents for NMR were purchased from Cambridge Isotope Laboratories, Inc. Eluents for column chromatography were HPLC grade and purchased from Fisher Scientific.

All reactions were performed open to the atmosphere, unless otherwise noted. Organic solutions were concentrated by use of a Buchi rotary evaporator. All polymerizations were carried out with temperature control under vacuum in flame-sealed ampoules. Chemical shifts are given in ppm relative to the signal from residual non-deuterated solvent. ^{1}H-NMR and ^{13}C-NMR spectra were recorded in $CDCl_3$ (except where noted) on Bruker DRX-300, DRX-400 or DRX-500 spectrometers. Data for ^{1}H NMR are reported as follows: chemical shift (δ ppm), multiplicity (s = singlet, br s = broad singlet, d = doublet, t = triplet, dd = doublet of doublets, dt = doublet of triplets, q = quartet, hept = heptet, m = multiplet), coupling constant (Hz), integration, and assignment. Data for ^{13}C are reported in terms of chemical shift. High-resolution mass spectra were obtained from the Columbia University Mass Spectrometry Facility on a JEOL JMSHX110 HF mass spectrometer using FAB+ ionization mode. Low-resolution mass spectrometry (LRMS) was performed on a JEOL JMS-LCmate liquid chromatography spectrometer system using APCl+ ionization technique.

Thin layer chromatography (TLC) was performed using Teledyne Silica gel 60 F254 plates and viewed under UV light. Flash column chromatography was performed using Teledyne Ultra Pure Silica Gel (230 – 400 mesh) on a Teledyne Isco Combiflash Rf.

Supplementary Methods

Size Exclusion Chromatography (SEC)

PCPMo was characterized to quantify its molecular mass dispersity (*Đ*) on a Waters Alliance 2695 separation module equipped with a PL-aquagel-OH 8 micron Mixed-M column (300 x 7.5 mm), a Waters 2998 Photodiode Array Detector, and a Waters 2414 Refractrometer Detector. Sodium acetate buffer (0.3 M) with 20 vol% methanol was used as the eluent at a flow rate of 0.7 mL min^{-1}. Poly(ethylene glcyol) standards were used for calibration.

Thermogravimetric Analysis (TGA)

Thermogravimetric analysis was performed on a Perkin-Elmer Pyris 1 TGA from ambient temperature to 600 °C at a rate of 10 °C min^{-1}. Polymer samples were dried under high vacuum overnight prior to measurement, and decomposition temperatures were recorded at 5% mass loss.

Differential Scanning Calorimetry (DSC)
Differential Scanning Calorimetry (DSC) was performed on a TA Instruments DSC Q2000 fitted with a RCS90 refrigerated cooling system to determine the glass transition temperatures. DSC measurements were taken at a sampling rate of 10 °C min^{-1} in the temperature range of 0 °C to 200 °C.
Dynamic Light Scattering (DLS)
Particle size, polydispersity, and electrophoretic mobility were measured using a Möbiuζ dynamic light scattering instrument and Dynamics software from Wyatt Technology (Santa Barbara, CA). Particle size and polydispersity were calculated via the Regularization fit of the correlation function of the Quasi-elastic Light Scattering (QELS) data. Each measurement contained 10 acquisitions and at least 3 measurements were performed. The reported radii or diameters are the average of those measurements. Zeta potential was calculated according to the Smoluchowski approximation and reported values are the averaged result of 5 acquisitions from each of the 31 detectors in the Massively Parallel Phase Amplitude Light Scattering (MP-PALS) detector array. Measurements were run in MilliQ water at neutral pH unless otherwise noted. Samples were passed through a 1.6 μm glass filter (Whatman) prior to measurement to remove only large aggregates and dust.

Scanning Electron Microscopy (SEM)
Scanning electron microscopy (SEM) was performed on a JEOL7001FLV at 3.0 to 10.0 keV. Particles were deposited on a silica wafer from solution, and imaged without sputter coating. Particle sizes measured by SEM were determined using Image-J software by manually counting at least 50 particles.

Quantification of water uptake
Water uptake of the polymer membrane was measured in a humidity-controlled environmental chamber (Espec). A small piece of water-equilibrated membrane was placed in a quartz pan which was hooked on the end of a quartz spring (Deerslayer) in the humidity chamber. The membrane was equilibrated at room temperature at 90% relative humidity for 48 hr. The mass of the hydrated film was obtained by measuring spring length through a port on the wall of the humidity chamber by a cathetometer equipped with an optical zoom telescope located outside the chamber. Care was taken to minimize the time when the port was opened (typically 10 s). The spring was calibrated with standard masses at experimental temperature and relative humidity in the chamber before use (spring constant was about 0.5 mN mm^{-1}). Dry mass of humid air-equilibrated membrane was measured following the same procedure as described above. The degree of hydration, λ_w, defined as the moles of water per mole of cationic groups in the membrane, is calculated using equation (1):

$$\lambda_{\mathrm{w}} = \frac{[H_2O]}{[CP]} = \frac{\text{hydrated film weight-dry film weight}}{\text{dry film weight}} \times \frac{M_{CP} + \left(x_{CP}^{-1} - 1\right)M_S}{M_W} \quad (1)$$

where the molar mass of water and of the styrene (S) and cyclopropenium (CP) monomers are M_{W} = 18.02 g mol^{-1}, M_{S} = 104.15 g mol^{-1} and M_{CP} = 419 g mol^{-1}.

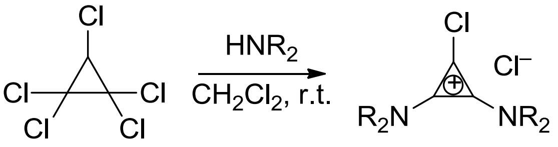

Procedures for synthesis of 2,3-bis(dialkylamino)-1-chlorocyclopropenium chloride

Synthesis of 2,3-bis(dicyclohexylamino)-1-chlorocyclopropenium chloride

Dicyclohexylamine (168 mL, 804.8 mmol, 6.0 equiv) was slowly added to a solution of pentachlorocyclopropane[1] (30.0 g, 140.0 mmol, 1.0 equiv) in CH_2Cl_2 (1500 mL) in a 3L round bottom flask. A white precipitate formed as the reaction mixture was stirred for a further 48 hr at room temperature. The solution was washed with 1M HCl (3 x 500 mL), dried with anhydrous sodium sulfate, and concentrated *in vacuo* to yield an off-white solid. This solid was triturated with hot ethyl acetate to give the title product (60 g, 130 mmol, 92%). ^{1}H NMR (500 MHz, $CDCl_3$) δ 3.75 (m, 2H, NCy**H**), 3.40 (m, 2H, NCy**H**), 1.10-2.20 (m, 40H, Cy**H**). ^{13}C NMR (125 MHz, $CDCl_3$) δ 131.6, 93.1, 65.2, 56.3, 32.1, 30.2, 24.9, 24.7, 24.0, 23.8. HRMS (FAB+) m/z = 431.3418 calcd for $C_{27}H_{44}N_2Cl$ $[M]^+$ 431.32.

Synthesis of 2,3-bis(diisopropylamino)-1-chlorocyclopropenium chloride

Pentachlorocyclopropane (5.20 g, 22.8 mmol, 1.0 equiv) was added to 230 mL of CH_2Cl_2 in a 500 mL dry round bottom flask equipped with a stir bar. To this solution, diisopropylamine (18.48 g, 182.6 mmol, 8.0 equiv) was slowly added and allowed to stir under argon at room temperature overnight. Solvent was removed from the reaction mixture leaving a crude, brown sandy-looking mixture of the desired product in quantitative yield and 3-4 equivalents of the corresponding ammonium salt. This crude mixture was used in subsequent steps without further purification. ^{1}H NMR (400 MHz, $CDCl_3$) δ 4.28 (hept, *J* = 6.7 Hz, 2H, C_3(NC**H**$(CH_3)_2$CH$(CH_3)_2)_2$), 3.89 (hept, *J* = 6.8 Hz, 2H, C_3(NCH$(CH_3)_2$C**H**$(CH_3)_2)_2$), 1.45 (m, 24H, C_3(N(CH(C**H**$_3)_2)_2)_2$). ^{13}C NMR (125 MHz, $CDCl_3$) δ 132.0, 117.8, 93.3, 58.1, 48.6, 47.1, 22.6, 21.8, 20.9, 18.9.

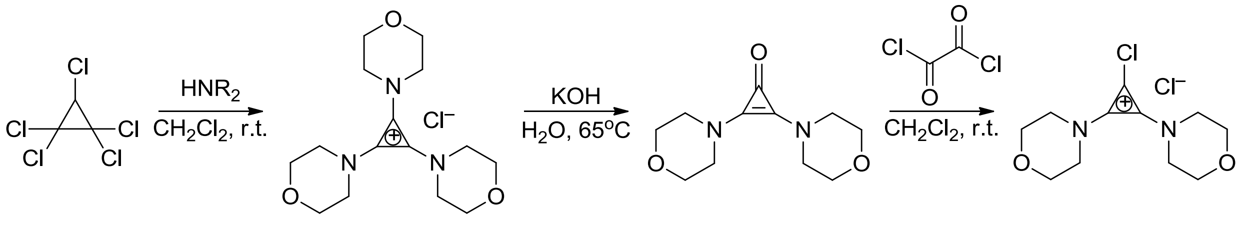

Synthesis of 2,3-bis(morpholino)-1-cyclopropenone

Morpholine (58.0 g, 665.7 mmol, 7.1 equiv) was slowly added to a solution of pentachlorocyclopropane (20.0 g, 93.3 mmol, 1.0 equiv) in CH_2Cl_2 (250 mL) in a 500 mL round bottom flask. The solution turned orange, and a white precipitate formed as the reaction mixture was stirred overnight at room temperature. The white solid was filtered off and the filtrate was concentrated *in vacuo* to a crude red solid. Water (100 mL) was used to dissolve this solid. A room temperature solution of potassium hydroxide (20 g, 356.4 mmol) in water (30 mL) was added to the solution, which was heated to 65 ºC for one hr. The reaction solution was allowed to

cool and water was then removed by rotary evaporation. The resulting solid was washed with CH_2Cl_2 (500 mL) and any remaining solid was filtered off. The organic solution was dried with anhydrous sodium sulfate and concentrated *in vacuo* to yield a crude orange solid. The crude material was purified by silica gel chromatography (10% MeOH in EtOAc) to yield the title product as an off-white solid (8.9 g, 39.7 mmol, 42% two-step yield). Note: the temperature of the rotovap was kept at 30 ºC or cooler, and extended exposure to methanol will decompose the title product. ^{1}H NMR (400 MHz, $CDCl_3$) δ 3.73 (m, 8H, $NCH_2C\mathbf{H_2}O$), 3.34 (m, 8H, $NC\mathbf{H_2}CH_2O$). ^{13}C NMR (125 MHz, $CDCl_3$) δ 134.8, 120.3, 66.1, 49.3. HRMS (FAB+) m/z = 225.1243 calcd for $C_{11}H_{17}N_2O_3$ $[M]^+$ 225.12.

Synthesis of 2,3-bis(morpholino)-1-chlorocyclopropenium chloride

Oxalyl chloride (6.86 mL, 79.4 mmol, 2.0 equiv) was slowly added to a 0 ºC solution of 2,3-bis(morpholino)-1-cyclopropenone (8.9 g, 39.7 mmol, 1.0 equiv) in CH_2Cl_2 (250 mL) under argon. The solution was warmed to room temperature and left to react for one hr. The product was dried *in vacuo* to yield a sufficiently pure black solid in quantitative yield (11.0 g, 39.7 mmol). ^{1}H NMR (400 MHz, $CDCl_3$) δ 4.05 (m, 4H, $N(HCH\text{-}CH\mathbf{H})_2O$), 3.90 (dt, *J* = 20.2, 4.5 Hz, 8H, $N(HC\mathbf{H}\text{-}C\mathbf{H}H)_2O$) 3.66 (m, 4H, $N(\mathbf{H}CH\text{-}CHH)_2O$).

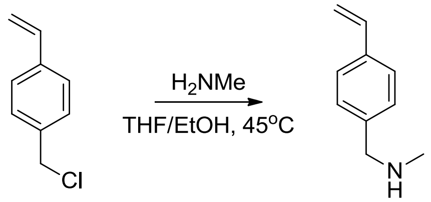

Synthesis of *N*-methyl-4-vinylbenzylamine

Vinylbenzyl chloride (7.5 g, 49.3 mmol, 1 equiv) was added to a 1L round bottom flask equipped with a stir bar. Methylamine solution (8.0M in ethanol, 101.1 mL, 15 equiv) was added to the sealed flask, and an outlet was used to relieve pressure. THF (330 mL) was added to dilute the reaction mixture such that the concentration of vinylbenzyl chloride was 0.10M. The flask was filled with argon and sealed with a septum secured with copper wire. The contents of the reaction flask were allowed to stir at 45 °C for 24 hr. Solvent was subsequently removed by rotary evaporation, and the crude product was dissolved in 250 mL of CH_2Cl_2 and transferred to a 1L separatory funnel. This solution was washed 3x with 1.0M NaOH, 1x with DI water, and 1x with brine, and dried over magnesium sulfate. Removal of solvent by rotary evaporation yielded the title product as yellow oil. ^{1}H NMR was used to determine purity. (6.82 g, 46.4 mmol, 94% yield, 90% purity). The oil was stored at 0 ºC and was used without further purification. ^{1}H NMR (400 MHz, $CDCl_3$) δ 7.33 (m, 4H, Ar**H**), 6.71 (dd, *J* = 17.6, 10.9 Hz, 1H, $H_2C{=}C\mathbf{H}Ar$), 5.73 (dd, *J* = 17.6, 1.0 Hz, 1H, $\mathbf{H_2}C{=}CHAr$), 5.21 (dd, *J* = 10.9, 2.3 Hz, 1H**,** $\mathbf{H_2}C{=}CHAr$), 3.73 (s, 2H, $ArC\mathbf{H_2}N$), 2.45 (s, 3H, $NC\mathbf{H_3}$).

Procedures for synthesis of CPR monomers

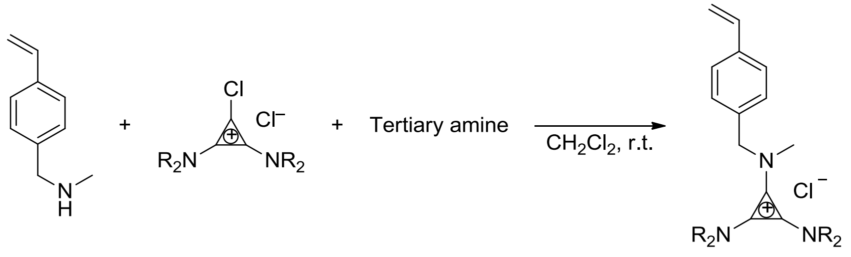

Characterization of *N*-methyl-1-(2,3-bis(dicyclohexylamino)cyclopropenium)-4-vinylbenzylamine chloride (CPCy). (See main text for synthetic protocol). ^{1}H NMR (500 MHz, $CDCl_3$) δ 7.33 (m, 4H, Ar**H**), 6.69 (dd, *J* = 17.7, 10.9 Hz, 1H, H_2C=C**H**Ar), 5.74 (d, *J* = 17.6 Hz, 1H, **H**$_2$C=CHAr), 5.25 (d, *J* = 11.0 Hz, 1H, **H**$_2$C=CHAr), 4.80 (s, 2H, ArC**H**$_2$N), 3.35 (m, 4H, NCy**H**), 3.22 (s, 3H, NC**H**$_3$), 1.00-1.90 (m, 40H, Cy**H**). ^{13}C NMR (125 MHz, $CDCl_3$) δ 137.4, 135.6, 133.6, 126.8, 126.6, 119.0, 117.8, 114.3, 60.3, 57.5, 39.4, 31.7, 25.2, 24.2. HRMS (FAB+) m/z = 542.4333 calcd for $C_{37}H_{56}N_3$ $[M]^+$ 542.45.

Synthesis of *N*-methyl-1-(2,3-bis(diisopropylamino)cyclopropenium)-4-vinylbenzylamine chloride (CPiP). In a 1 L round bottom flask equipped with stir bar, 2,3-bis(diisopropylamino)-1-chlorocyclopropenium chloride (26.23 g crude mixture, 38.0 mmol CP salt, 1.0 equiv) and triethylamine (11.5 g, 114.0 mmol, 3.0 equiv) were dissolved in 420 mL of CH_2Cl_2 and put under an atmosphere of argon. *N*-Methyl-4-vinylbenzylamine (5.59 g, 38.0 mmol, 1.0 equiv) was slowly added to the solution, which was stirred for 15 hr. The reaction mixture was poured into a 1 L separatory funnel and washed with 1M HCl (3 x 200 mL), then DI water (1 x 200 mL), followed by brine (1 x 200 mL). The organic layer was collected and dried over magnesium sulfate. Rotary evaporation yielded 20.0 g of a dark brown viscous liquid. This crude product was purified by silica gel chromatography, eluted first with 100% EtOAc, followed by a mixture of 5% increasing to 20% MeOH in CH_2Cl_2. Collection of pure fractions, followed by removal of solvent by rotary evaporation yielded an amber oil (13.97 g, 33.3 mmol, 88% yield) ^{1}H NMR (400 MHz, $CDCl_3$) δ 7.44 (m, 2H, Ar**H**), 7.25 (m, 2H, Ar**H**), 6.69 (dd, *J* = 17.6, 10.9 Hz, 1H, H_2C=C**H**Ar), 5.78 (dd, *J* = 17.6, 0.8 Hz, 1H, **H**$_2$C=CHAr), 5.29 (dd, *J* = 10.9, 0.8 Hz, 1H **H**$_2$C=CHAr), 4.82 (s, 2H, ArC**H**$_2$N), 3.90 (hept, 4H, C_3NC**H**(Me)$_2$), 3.24 (s, 3H, NC**H**$_3$), 1.00-1.90 (m, 24H, NCH(C**H**$_3$)$_2$). ^{13}C NMR (125 MHz, $CDCl_3$) δ 137.7, 135.9, 134.0, 127.1, 126.9, 119.0, 116.9, 114.6, 57.9, 51.5, 39.8, 22.01. HRMS (FAB+) m/z = 382.3241 calcd for $C_{25}H_{40}N_3$ $[M]^+$ 382.32.

Synthesis of *N*-methyl-1-(2,3-Bis(morpholino)cyclopropenium)-4-vinylbenzylamine chloride (CPMo). To a dry round bottom flask of 2,3-bis(morpholino)-1-chlorocyclopropenium chloride (11.08 g, 39.7 mmol, 1.0 equiv) under argon was added CH_2Cl_2 (150 mL) and *N*,*N*-diethylmethylamine (5.3 mL, 43.7 mmol, 1.1 equiv). *N*-Methyl-4-vinylbenzylamine (5.3 g, 35.7 mmol, 0.9 equiv) was then slowly added to solution, and the reaction was left overnight. The crude product was concentrated *in vacuo* and dissolved in 250 mL of $CHCl_3$:iPrOH (2:1). The solution was extracted with water (2 x 100 mL), dried with anhydrous sodium sulfate, and concentrated *in vacuo* to yield a crude solid. A portion of the product is lost in the aqueous wash. The crude product was purified with silica gel chromatography (20% MeOH in CH_2Cl_2) to yield a dark solid (10.0 g, 25.6 mmol, 59% yield). ^{1}H NMR (500 MHz, $CDCl_3$) δ 7.44 (m, 2H, Ar**H**),

7.24 (m, 2H, Ar**H**), 6.70 (dd, *J* = 17.7, 11.0 Hz, 1H, H_2C=C**H**Ar), 5.76 (dd, *J* = 17.9, 1.7 Hz, 2H, **H**$_2$C=CHAr), 5.28 (dd, *J* = 10.9, 1.5 Hz, 2H, **H**$_2$C=CHAr), 4.65 (s, 2H, ArC**H**$_2$N), 3.81 (m, 8H, NC**H**$_2$CH$_2$O), 3.54 (m, 8H, NCH$_2$C**H**$_2$O), 3.21 (s, 3H, NC**H**$_3$). ^{13}C NMR (125 MHz, $CDCl_3$) δ 137.8, 135.9, 133.8, 127.0, 127.0, 118.8, 117.1, 114.7, 65.8, 58.6, 50.0, 40.8. HRMS (FAB+) m/z = 354.2171 calcd for $C_{21}H_{28}N_3O_2$ $[M]^+$ 354.22.

Procedures for RAFT homopolymerizations of PCPR

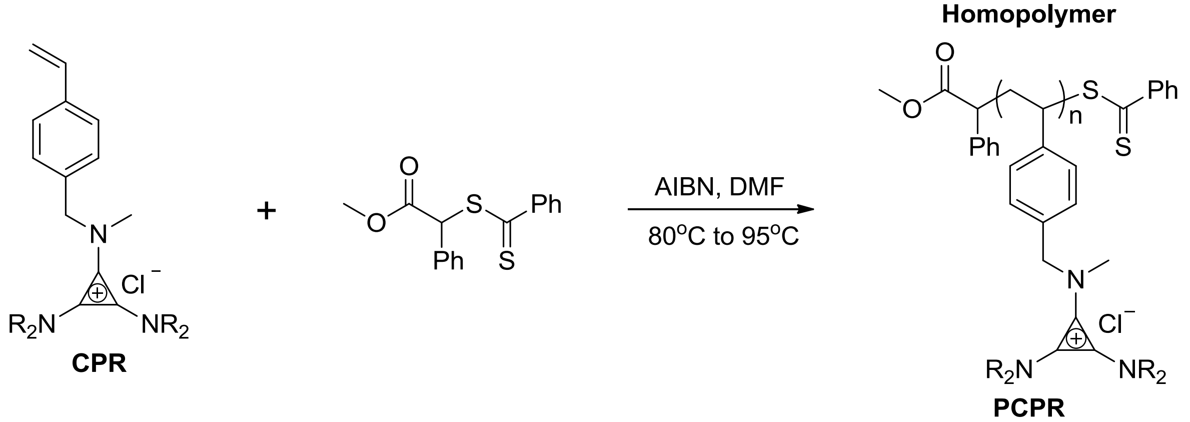

Characterization of PCPCy. (See main text for synthetic protocol). ^{1}H NMR (500 MHz, $CDCl_3$) δ 7.76-7.60 (b, 2H, -SC(Ar**H**)S-), 7.51-6.00 (b, 240H, Ar**H**), 5.17-4.58 (b, 120H, ArC**H**$_2$N), 3.61-2.94 (b, 420H, NCy**H**, NC**H**$_3$), 2.05-0.75 (b, 2580H, Cy**H**, ArC**H**C**H**$_2$).

Synthesis of PCPiP. **CPiP** (3.5 g, 83.1 mmol, 50.0 equiv), MCPDB (50.2 mg, 1.66 mmol, 1.0 equiv), AIBN (.54 mg, 0.033 mmol, 0.20 equiv), and *N,N*-dimethylformamide (DMF) (0.60 mL) were added to a flame seal ampoule and vortexed to form a homogenous solution. A stir bar was added to the ampoule, and after 4 freeze-pump-thaw cycles to remove oxygen, the ampoule was sealed under vacuum. The polymerization was run for 2 hr 15 min at 100 ºC. The reaction mixture was precipitated from CH_2Cl_2 into –78 °C ethyl acetate 5 times to remove monomer. Drying *in vacuo* yielded the polymer as a pink powder (2.45 g, 70% yield). Alternatively, the reaction mixture could be transferred to a 3.5k MWCO Spectrum labs dialysis bag to dialyze for 24 hr in 1L of water. ^{1}H NMR (500 MHz, $CDCl_3$) δ 7.06-6.45 (b, 166H, Ar**H**), 4.98-4.65 (b, 80H, ArC**H**$_2$N), 3.98-3.79 (b, 165H, C_3NC**H**(iPr)$_2$), 3.48 (s, 3H, OC**H**$_3$), 1.67-1.27 (b, 1700H, iPr**H**, ArC**H**C**H**$_2$).

Synthesis of PCPMo. To a dry 20 mL scintillation vial, **CPMo** (7.0 g, 17.9 mmol, 700 equiv), MCPDB (54 mg, 0.179 mmol, 1.0 equiv), AIBN (4.4 mg, 0.0269 mmol, 0.15 equiv), and DMF (7.0 mL) were added and vortexed to form a homogenous solution. This solution was transferred to a flame-dried ampoule. After 4 freeze-pump-thaw cycles, the ampoule was sealed under vacuum. The polymerization was run for 12 hr at 85 ºC. The reaction mixture was then transferred into a 3.5k MWCO Spectrum labs dialysis bag and left to dialyze for 24 hr in 1L of water. The water was changed five times in this time. The resulting polymer solution was freeze-dried to yield the pure polymer as a brown solid (3.6 g, 51% yield). ^{1}H NMR (500 MHz, CD_3OD) δ 7.90-7.74 (b, 2H, -SC(Ar**H**)S-), 7.67-6.26 (b, 300H, Ar**H**), 4.77-4.46 (b, 150H, ArC**H**$_2$N), 3.87-3.62 (b, 600H, NC**H**$_2$CH$_2$O), 3.62-3.38 (b, 600H, NCH$_2$C**H**$_2$O), 3.29-3.03 (b, 225H, NC**H**$_3$), 2.55-1.04 (b, 225H, ArC**H**C**H**$_2$).

Procedures for RAFT Block Polymerization of PS-b-PCPR

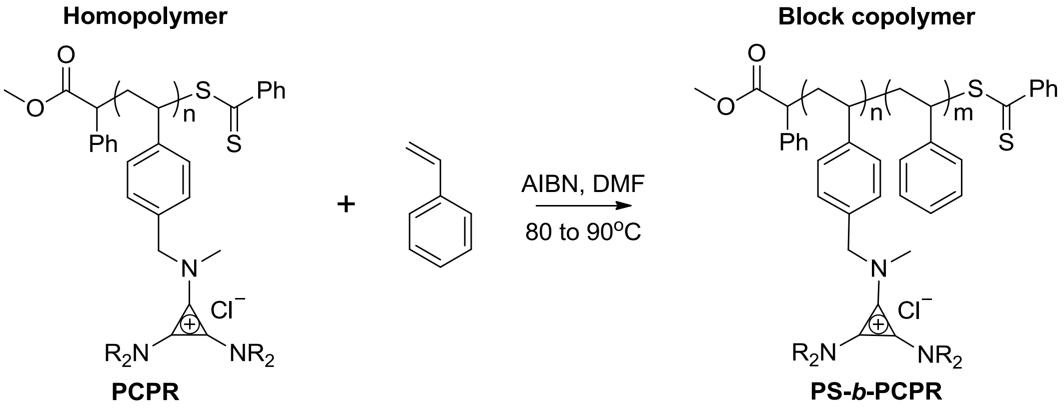

Synthesis of PS-*b*-PCPCy(45). To a dry 20 mL scintillation vial, **PCPCy** (1.0 g, 0.029 mmol, 1.0 equiv), AIBN (7.0 mg, 4.3 mmol, 0.15 equiv), styrene (0.722 g, 6.93 mmol, 4 equiv), and DMF (1.75 mL) were added and vortexed to form a homogenous solution. This solution was transferred to a flame-dried ampoule. After 4 freeze-pump-thaw cycles, the ampoule was sealed under vacuum. The polymerization was run for 12 hr at 80 ºC. The reaction mixture was precipitated from CH_2Cl_2 into hexanes 3 times. Drying *in vacuo* yielded the pure polymer as a pink powder (0.850 g, 70% yield). ^{1}H NMR (500 MHz, $CDCl_3$) δ 7.90-7.78 (b, 2H, -SC(Ar**H**)S-), 7.51-6.04 (b, 600H, Ar**H**), 5.23-4.58 (b, 120H, ArC**H₂**N), 3.73-2.96 (b, 420H, NCy**H**, NC**H₃**), 2.05-0.75 (b, 2800H, Cy**H**, ArC**HCH₂**).

Synthesis of PS-*b*-PCPCy(30). To a dry 20 mL scintillation vial, **PCPCy** (1.0 g, 0.029 mmol, 1.0 equiv), AIBN (7.0 mg, 4.3 mmol, 0.15 equiv), styrene (1.08 g, 10.4 mmol, 6 equiv), and DMF (1.75 mL) were added and vortexed to form a homogenous solution. This solution was transferred to a flame-dried ampoule. After 4 freeze-pump-thaw cycles, the ampoule was sealed under vacuum. The polymerization was run for 12 hr at 80 ºC. The reaction mixture was precipitated from CH_2Cl_2 into hexanes 3 times. Drying *in vacuo* yielded the pure polymer as a pink powder (0.850 g, 60% yield). ^{1}H NMR (500 MHz, $CDCl_3$) δ 7.90-7.78 (b, 2H, -SC(Ar**H**)S-), 7.51-6.04 (b, 940H, Ar**H**), 5.23-4.58 (b, 120H, ArC**H₂**N), 3.73-2.96 (b, 420H, NCy**H**, NC**H₃**), 2.05-0.75 (b, 3180H, Cy**H**, ArC**HCH₂**).

Synthesis of PS-*b*-PCPiP(50). To a dry flame-seal ampoule with stir bar, **PCPiP** (226.5 mg, 0.012 mmol, 1.0 equiv), AIBN (0.44 mg, 0.0027 mmol, 0.2 equiv), styrene (0.277 g, 2.66 mmol, 200 equiv), and DMF (0.130 mL) were added and vortexed to form a homogenous solution. After 4 freeze-pump-thaw cycles, the ampoule was sealed under vacuum. The polymerization was stirred vigorously for 8 hr at 100 °C. The reaction mixture was precipitated from CH_2Cl_2 into –78 °C ethyl acetate 3 times. Drying *in vacuo* yielded the pure polymer as a pale pink powder (0.240 g, 88% yield). ^{1}H NMR (500 MHz, $CDCl_3$) δ 7.07-6.46 (b, 314H, Ar**H**), 5.11-4.59 (b, 80H, ArC**H₂**N), 4.11-3.74 (b, 172H, C_3NC**H**(iPr)₂), 3.93-2.97 (b, 122H, NC**H₃**), 2.30-0.98 (b, 1600H, iPr**H**, ArC**HCH₂**).

Synthesis of PS-*b*-PCPiP(30). To a dry flame-seal ampoule with stir bar, **PCPiP** (271 mg, 0.014 mmol, 1.0 equiv), AIBN (0.52 mg, 0.0032 mmol, 0.2 equiv), styrene (0.414 g, 3.99 mmol, 250 equiv), and DMF (0.240 mL) were added and vortexed to form a homogenous solution. After 4 freeze-pump-thaw cycles, the ampoule was sealed under vacuum. The polymerization was stirred vigorously for 24 hr at 100 °C. The reaction mixture was precipitated from CH_2Cl_2 into –78 ºC ethyl acetate 3 times. Drying *in vacuo* yielded the pure polymer as a pale pink powder (0.310 g, 65% yield). ^{1}H NMR (500 MHz, $CDCl_3$) δ 7.26-6.27 (b, 510H, Ar**H**), 4.90-4.59 (b, 80H, ArC**H₂**N), 4.01-3.71 (b, 167H, C_3NC**H**(iPr)$_2$), 3.34-2.95 (b, 118H, NC**H₃**), 2.10-1.10 (b, 1690H, iPr**H**, ArC**H**C**H₂**).

Synthesis of PS-*b*-PCPiP(20). To a dry flame-seal ampoule with stir bar, **PCPiP** (230 mg, 0.012 mmol, 1.0 equiv), AIBN (0.2 mg, 0.0012 mmol, 0.1 equiv), styrene (1.37 g, 13.2 mmol, 1000 equiv), and DMF (0.460 mL) were added and vortexed to form a homogenous solution. After 4 freeze-pump-thaw cycles, the ampoule was sealed under vacuum. The polymerization was stirred vigorously for 30 hr at 95 ºC. Such a large excess of styrene was used so the polymer would not precipitate out of solution during the reaction. The reaction mixture was precipitated from CH_2Cl_2 into a –78 ºC solution of 25% ethyl acetate in hexanes 3 times. Drying *in vacuo* yielded the pure polymer as a pale pink powder (0.270 g, 62% yield). ^{1}H NMR (400 MHz, $CDCl_3$) δ 7.26-6.24 (b, 900H, Ar**H**), 4.95-4.59 (b, 80H, ArC**H₂**N), 4.01-3.77 (b, 176H, C_3NC**H**(iPr)$_2$), 3.27-3.01 (b, 126H, NC**H₃**), 2.02-0.94 (b, 1590H, iPr**H**, ArC**H**C**H₂**).

Synthesis of PS-*b*-PCPMo(50). To a dry 20 mL scintillation vial, **PCPMo** (0.700 g, 0.024 mmol, 1.0 equiv), AIBN (0.59 mg, 0.0036 mmol, 0.15 equiv), styrene (0.75 g, 7.2 mmol, 4 equiv), and DMF (2.5 mL) were added and vortexed to form a homogenous solution. The large volume of DMF was necessary to totally dissolve **PCPMo**. This solution was transferred to a flame-dried ampoule. After 4 freeze-pump-thaw cycles, the ampoule was sealed under vacuum. The polymerization was run for 12 hr at 85 ºC. The reaction mixture was precipitated from CH_2Cl_2 into diethyl ether 2 times. Drying *in vacuo* yielded the pure polymer as a pink powder (0.820 g, 90% yield). ^{1}H NMR (500 MHz, $(CD_3)_2SO$) δ 7.86-7.71 (b, 2H, -SC(Ar**H**)S-), 7.47-6.09 (b, 760H, Ar**H**), 4.90-4.28 (b, 150H, ArC**H₂**N), 3.85-3.54 (b, 600H, NC**H₂**CH$_2$O), 3.54-3.30 (b, 600H, NCH$_2$C**H₂**O), 3.22-2.86 (b, 225H, NC**H₃**), 2.15-1.12 (b, 500H, ArC**H**C**H₂**).

Synthesis of PS-*b*-PCPMo(35). To a dry 20 mL scintillation vial, **PCPMo** (0.900 g, 0.031 mmol, 1.0 equiv), AIBN (0.76 mg, 0.0046 mmol, 0.15 equiv), styrene (2.3 g, 22.2 mmol, 10 equiv), and DMF (8.0 mL) were added and vortexed to form a homogenous solution. The large volume of DMF was necessary to totally dissolve **PCPMo**. This solution was transferred to a flame-dried ampoule. After 4 freeze-pump-thaw cycles, the ampoule was sealed under vacuum. The polymerization was run for 12 hr at 85 ºC. The reaction mixture was precipitated from CH_2Cl_2 into diethyl ether 2 times. Drying *in vacuo* yielded the pure polymer as a pink powder (1.19 g, 88% yield). ^{1}H NMR (500 MHz, $(CD_3)_2SO$) δ 7.86-7.71 (b, 2H, -SC(Ar**H**)S-), 7.58-6.05 (b, 1000H, Ar**H**), 5.01-4.28 (b, 150H, ArC**H₂**N), 3.85-3.53 (b, 600H, NC**H₂**CH$_2$O), 3.53-3.24 (b, 600H, NCH$_2$C**H₂**O), 3.21-2.57 (b, 225H, NC**H₃**), 2.23-1.08 (b, 640H, ArC**H**C**H₂**).

Synthesis of PS-*b*-PCPMo(30). To a dry 20 mL scintillation vial, **PCPMo** (0.900 g, 0.031 mmol, 1.0 equiv), AIBN (0.76 mg, 0.0046 mmol, 0.15 equiv), styrene (4.0 g, 38.9 mmol, 17 equiv), and DMF (11.25 mL) were added and vortexed to form a homogenous solution. The

large volume of DMF was necessary to totally dissolve **PCPMo**. This solution was transferred to a flame-dried ampoule. After 4 freeze-pump-thaw cycles, the ampoule was sealed under vacuum. The polymerization was run for 12 hr at 85 ºC. The reaction mixture was precipitated from CH_2Cl_2 into diethyl ether 2 times. Drying *in vacuo* yielded the pure polymer as a pink powder (1.19 g, 82% yield). ^{1}H NMR (500 MHz, $(CD_3)_2SO$) δ 7.86-7.71 (b, 2H, -SC(Ar**H**)S-), 7.47-6.03 (b, 1175H, Ar**H**), 4.92-4.30 (b, 150H, ArC$\mathbf{H_2}$N), 3.81-3.54 (b, 600H, NC$\mathbf{H_2}$CH$_2$O), 3.54-3.34 (b, 600H, NCH$_2$C$\mathbf{H_2}$O), 3.21-2.92 (b, 225H, NC$\mathbf{H_3}$), 2.21-0.92 (b, 750H, ArC**H**C$\mathbf{H_2}$).

Procedures for RAFT random copolymerization of P(S-r-CPR)

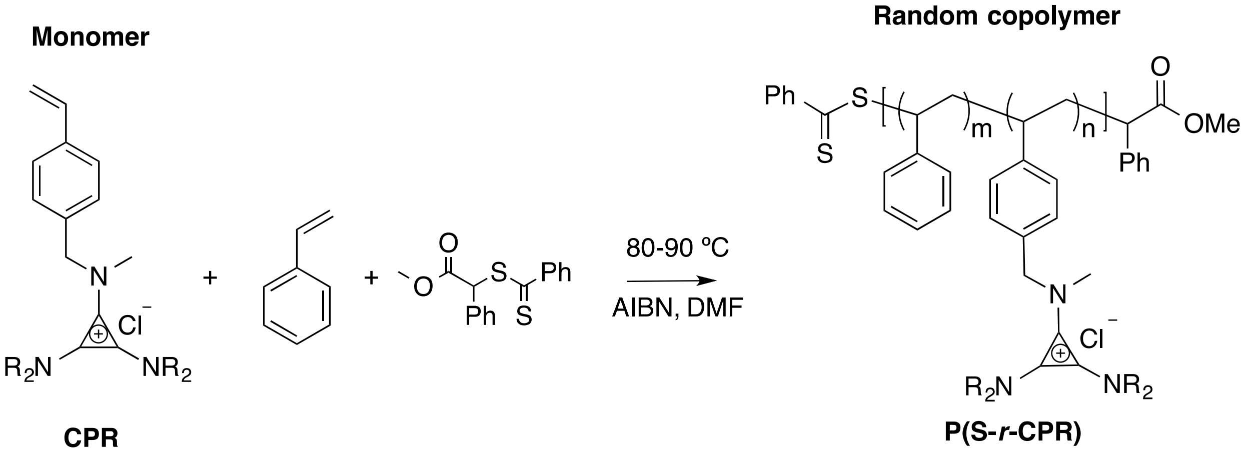

Synthesis of P(S-*r*- CPCy). To a dry 20 mL scintillation vial, **CPCy** (1.00 g, 1.73 mmol, 50.0 equiv), styrene (0.180 g, 1.73 mmol, 50.0 equiv), MCPDB (10.5 mg, 0.0346 mmol, 1.0 equiv), AIBN (0.852 mg, 0.00519 mmol, 0.15 equiv), and DMF (0.500 mL) were added and vortexed to form a homogenous solution. This solution was transferred to a flame-dried ampoule. After 4 freeze-pump-thaw cycles, the ampoule was sealed under vacuum. The polymerization was run for 12 hr at 80 ºC. The reaction mixture was precipitated three times into ethyl acetate and once in hexanes. Drying *in vacuo* yielded the pure polymer as a pink powder (790 mg, 67% yield). Integration of the ^{1}H NMR showed approximately 35 units of **CPCy** and 35 units of styrene (50% **CPCy** incorporation). ^{1}H NMR (500 MHz, $CDCl_3$) δ 7.89-7.69 (b, 2H, -SC(Ar**H**)S-), 7.39-6.04 (b, 600H, Ar**H**), 5.09-4.42 (b, 120H, ArC$\mathbf{H_2}$N), 3.60-2.89 (b, 420H, NCy**H**, NC$\mathbf{H_3}$), 2.09-0.77 (b, 2800H, Cy**H**, ArC**H**C$\mathbf{H_2}$).

Synthesis of P(S-*r*-CPiP). To a dry, flame-seal ampoule with stir bar, **CPiP** (1.8 g, 4.24 mmol, 50 equiv), styrene (0.446 g, 4.29 mmol, 50 equiv), AIBN (1.4 mg, 0.0086 mmol, 0.1 equiv), 2-cyanopropan-2-yl benzodithioate (19 mg, 0.086, 1.0 equiv) and DMF (0.233 mL) were added and vortexed to form a homogenous solution. After 4 freeze-pump-thaw cycles, the ampoule was sealed under vacuum. The polymerization was stirred vigorously for 17 hr at 95 ºC. The reaction mixture was precipitated from CH_2Cl_2 into a –78 ºC solution of 25% ethyl acetate in hexanes 3 times. Drying *in vacuo* yielded a pale pink powder composed of approximately 13 units of **CPiP** and 15 units of styrene (0.270 g, 62% yield, 47% incorporation of **CPiP**). ^{1}H NMR (400 MHz, $CDCl_3$) δ 7.26-6.24 (b, 920H, Ar**H**), 4.95-4.59 (b, 90H, ArC$\mathbf{H_2}$N), 4.01-3.77 (b, 200H, C_3NC**H**(iPr)$_2$, 3.27-3.01 (b, 136H, NC$\mathbf{H_3}$), 2.02-0.94 (b, 1750H, iPr**H**, ArC**H**C$\mathbf{H_2}$).

Synthesis of P(S-*r*-CPMo). To a dry 20 mL scintillation vial, **CPMo** (0.500 g, 1.28 mmol, 50.0 equiv), styrene (0.134 g, 1.28 mmol, 50.0 equiv), MCPDB (7.74 mg, 0.0256 mmol, 1.0 equiv), AIBN (0.632 mg, 0.00385 mmol, 0.15 equiv), and DMF (0.500 mL) were added and vortexed to form a homogenous solution. This solution was transferred to a flame-dried ampoule. After 4 freeze-pump-thaw cycles, the ampoule was sealed under vacuum. The polymerization was run for 12 hr at 90 ºC. The reaction mixture was precipitated once into –78 °C ethyl acetate to remove styrene. The precipitate was dissolved in water and transferred to a 1.0k MWCO Spectrum Labs dialysis bag and left to dialyze for 24 hr in 1L of water. The water was changed 5 times during this time. The resulting polymer was freeze-dried to yield pure polymer as a brown solid (0.190 g, 30% yield). Integration of the ^{1}H NMR showed about 25 units of **CPMo** and 30 units of styrene (45% **CPMo** incorporation). ^{1}H NMR (500 MHz, $(CD_3)_2SO$) δ 7.86-7.70 (b, 2H, -SC(Ar**H**)S-), 7.56-6.18 (b, 1175H, Ar**H**), 4.90-4.23 (b, 150H, ArC$\mathbf{H_2}$N), 3.93-3.53 (b, 600H, NC$\mathbf{H_2}$$CH_2$O), 3.53-3.17 (b, 600H, NCH_2C$\mathbf{H_2}$O), 3.17-2.85 (b, 225H, NC$\mathbf{H_3}$), 2.40-1.05 (b, 225H, ArC**H**C$\mathbf{H_2}$).

Procedure for Emulsion Polymerization

Synthesis of surfactant-free emulsion particles. Particles were synthesized by following a general procedure that was scaled accordingly using 1-20 wt.% **CPiP** (relative to styrene), styrene, 2,2′-azobis(2-methylpropionamidine) dihydrochloride (V-50), and water. The final solution was scaled to 10 grams, with 10 wt.% monomer content. First, **CPiP** was dissolved in styrene and initiator was dissolved separately in 1 mL of water. The remaining volume of water was added to the monomer solution, and the V-50 solution was finally added to the monomer suspension. The mixture was vortexed for 30 seconds. The solution was added to a two-neck flask fitted with a condenser and stirbar, and was sparged with N_2 for 10 minutes. The solution was stirred at 70°C for 6-16 hours.

Supplementary Reference

1 Tobey, S. W. & West, R. Pentachlorocyclopropane. *J. Am. Chem. Soc.* **88**, 2478-2481, (1966).
